# Regulation of hedonic feeding rhythms by circadian clocks in leptin-receptive neurons

**DOI:** 10.1016/j.molmet.2025.102221

**Published:** 2025-07-24

**Authors:** Jazmin Osorio-Mendoza, Jana-Thabea Kiehn, Sarah Stenger, Keno O. Heinen, Laura Griewahn, Christiane E. Koch, Undine Haferkamp, Violetta Pilorz, Johanna L. Barclay, Parth Joshi, Lisbeth Harder, Olaf Jöhren, Peter Kühnen, Gregor Eichele, Henrik Oster

**Affiliations:** 1 Institute of Neurobiology, University of Lübeck, Lübeck, Germany; 2 Center of Brain, Behavior & Metabolism, University of Lübeck, Lübeck, Germany; 3 Lübeck Institute of Experimental Dermatology, University of Lübeck, Lübeck, Germany; 4 Australian Alliance for Indigenous Genomics, Australia; 5 Max Planck Institute for Multidisciplinary Sciences, Göttingen, Germany; 6 Karolinska Institutet, Stockholm, Sweden; 7 Department of Pediatric Endocrinology and Diabetology, Charité Universitätsmedizin Berlin, Berlin, Germany

**Keywords:** Circadian clock, Leptin receptor, Feeding rhythms, Hedonic appetite

## Abstract

**Objective:**

The circadian clock anticipates daily repetitive events to adapt physiological processes. In mammals, the circadian system consists of a master clock in the suprachiasmatic nucleus (SCN), which synchronizes subordinate tissue clocks, including extra-SCN central nervous system (CNS) clocks involved in functions such as sleep and appetite regulation. Appetite is controlled by both homeostatic and non-homeostatic (hedonic) circuits. Homeostatic appetite addresses energy needs, while hedonic feeding targets cravings for palatable, calorie-dense foods. The adipokine leptin is a major appetite regulator, interacting with the circadian clock. Although leptin's role in satiation through its action in the mediobasal hypothalamus (MBH) is well established, its involvement in the circadian regulation of feeding remains poorly understood. We hypothesized that circadian gating of leptin signaling in the CNS controls homeostatic and hedonic appetite across the day.

**Methods:**

We analyzed food intake rhythms in mice with a loss of leptin (*ob/ob* mice) or clock function (*Per1/2* or *Bmal1 KO*) and in mice with specific disruption of leptin circadian gating in the CNS (*ObRb.Bmal1*).

**Results:**

We found that in leptin-deficient mice hedonic appetite increases specifically in the early rest phase. In contrast, clock-deficient *Per1/2* mutant mice exhibit blunted rhythms in both hedonic and homeostatic appetite control. Finally, when clock function is disrupted in leptin-sensitive neurons only, mice display a lower sensitivity to palatable food, along with reduced initial weight gain and adipose hypertrophy under obesogenic diet conditions.

**Conclusions:**

Our data describe a local clock-controlled central leptin gating mechanism that modulates hedonic food intake rhythms and impacts metabolic homeostasis.

## Highlights

• The adipokine leptin is a major appetite regulator, interacting with the circadian clock.
• Leptin-deficient mice show increased hedonic appetite specifically in the early rest phase.
• Circadian clock-deficient mice exhibit blunted rhythms in hedonic appetite control.
• Loss of Bmal1 in ObRb neurons leads to lower palatable food sensitivity and adipose hypertrophy under obesogenic diet conditions.
• A circadian leptin gating mechanism modulates hedonic food intake rhythms and impacts metabolic homeostasis.

## 1 Introduction

The global obesity pandemic is an increasing threat to human health and well-being. Since the 1970s, overweight and obesity rates have tripled [1], now affecting close to 40 % of the population worldwide [2,3]. Obesity is linked to an increased risk of cardiovascular and infectious diseases, diabetes mellitus type 2, infertility and miscarriages, and various other disorders [1,4,5]. It is rooted in an overconsumption of food, *i.e.*, nutrient intake beyond physiological requirements, due to dysregulated appetite and influenced by the interaction between the individual's genetic/epigenetic profile and environmental stimuli. Food intake is controlled by homeostatic and non-homeostatic regulatory circuits [6,7]. Homeostatic appetite serves to satisfy metabolic demands while non-homeostatic – or hedonic – appetite describes the craving for palatable, energy-dense sweet or savory foods largely independent of energetic needs. Homeostatic circuits that center on the MBH integrate metabolic signals from peripheral tissues such as liver, adipose tissue or the gastrointestinal tract [[7], [8], [9]]. Hedonic appetite regulation, in turn, engages dopaminergic circuits of the central reward system [10,11]. Palatable foods affect meso-limbic and meso-cortical dopamine signaling [7,12] similar to many drugs, promoting the motivation for further consumption and overriding homeostatic requirements [13,14].

An important metabolic signal affecting both homeostatic and hedonic aspects of satiety regulation is the adipokine hormone leptin [[15], [16], [17]]. Leptin is primarily produced by white adipose tissue (WAT) and signals high energy states [18]. In the MBH, it promotes satiety and increases energy expenditure via its cognate transmembrane receptor, ObR (or LepR) [8]. Six splice variants of ObR have been reported (ObRa-f) [19], of which only the long isoform, ObRb, contains the intracellular domains needed for the initiation of downstream signaling pathways [20]. In the brain, ObRb expression has been described in homeostatic regulatory nuclei of the MBH, but also in dopaminergic neurons of the Ventral tegmental area (VTA) and in the SCN, the master regulator of 24-hour, so-called circadian rhythms [21,22]. In the hypothalamus, leptin binding to ObRb leads to the activation of the Janus kinase 2/signal transducer and activator of transcription 3 (JAK2/STAT3) signaling [[23], [24], [25], [26]] and transcription of anorexigenic peptide genes such as *pro-opiomelanocortin* (*Pomc*) while inhibiting the expression of appetite-promoting peptides like agouti-related protein (AgRP) and neuropeptide Y (NPY) [[25], [26], [27]].

The circadian clock system comprises a ubiquitous network of cellular oscillators that regulates physiology and behavior to optimally align with environmental demands along the 24-hour day–night cycle. The molecular clock is based on transcriptional-translational feedback loops of clock genes such as *brain* and *muscle arnt-like 1* (*Arntl* or *Bmal1*), *circadian locomotor output cycles kaput* (*Clock*), *period* (*Per1-3*), and *cryptochrome* (*Cry1/2*) which exhibit pronounced circadian rhythms in mRNA and protein abundance [28,29]. In mammals, the main pacemaker of the circadian system resides in the hypothalamic SCN [30] which synchronizes with the external light–dark cycle via direct retinal projections [31]. Circadian rhythm disruption – through genetic manipulation or external perturbation of circadian rhythms such as jet lag and shift work – promotes overeating with elevated rest phase intake and increases the risk of developing obesity and other metabolic disorders [32,33].

In humans and rodents, leptin blood levels show diurnal rhythms regulated by interaction of feeding patterns and circadian clocks [[34], [35], [36], [37]]. Leptin deficiency in *ob/ob* mice, in turn, affects circadian regulation at a very young age, thus initiating a vicious cycle of metabolic dysfunction [33,38]. Recent data suggest that leptin affects homeostatic appetite regulation in the MBH [39], but the extent to which leptin contributes to the circadian rhythms of hedonic circuits remains unknown.

In this study, we show that the circadian modulation of central leptin signaling is regulated by clocks in ObRb-expressing neurons. Although the loss of clock function in these cells only modestly affects homeostatic feeding patterns, it blunts the circadian rhythms of palatable snack overconsumption. Our data suggest a central circadian gating mechanism residing in leptin-receptive neurons to control hedonically motivated overeating.

## 2 Material and methods

### 2.1 Animals

Leptin deficient (*ob/ob*) mice and heterozygous controls (*ob/+*) were used for measuring body weight development, homeostatic food and homeostatic/hedonic liquid consumption. *Per1/2* double mutant mice (*B6.Cg-Per1<tm1Brd> Tyr<c-Brd>*/*J x B6.Cg-Per2<tm1Brd> Tyr<c-Brd>/J*) [40] and congenic wildtype controls were used for analyzing the role of circadian clock function in appetite regulation. *ObRb.Bmal1* mice (homozygous mice, *B6.129-Leprtm2(cre)Rck/J x B6.129S4(Cg)-Arntltm1Weit/J*) carrying a deletion of the essential clock gene *Bmal1* – and, thus, clock function – in ObRb-positive neurons were generated by crossing *Bmal1-flox* animals [41] with mice expressing CRE recombinase as a knock-in into a long-isoform specific exon of the *ObR* gene [42]. CRE-positive/*Bmal1*^*wt/wt*^ was used as control. For mapping CRE-mediated recombination in the brain *ObRb.Bmal1* mice were crossed with ZsGreen (green fluorescent protein, GFP) reporter mice (*B6.Cg-Gt(ROSA)26Sortm6(CAG-ZsGreen1)Hze/J*) [41,42].

All mice were maintained on a C57BL/6J genetic background. Mice were kept under standard laboratory conditions: 22 ± 2 °C, 12-hour light: 12-hour dark (LD) cycle (*Zeitgeber* time (ZT) 0 = “lights on”), a relative humidity of 60 ± 5 % and unlimited access to standard chow (#1314, Altromin, Lage, Germany) and tap water if not otherwise stated. Experimental groups consisted of weight- and age-matched male mice, and all experiments were ethically approved by the Committees on Animal Health and Care of the State Governments of Schleswig–Holstein or Lower Saxony (4(132-10/13), 4(96-7/12), 4(08–30/17), V241-363122018(132-10/13) and performed in accordance with international ethical and experimental guidelines.

### 2.2 Homeostatic and hedonic food intake behavior

For the determination of **homeostatic feeding rhythms**, mice were individually housed and fed ad libitum with standard chow or high-fat experimental diet (HFD, 60 % fat; E−15742, Ssniff, Soest, Germany) in LD. **Comparative daily profiles of food intake** were determined at 6-hour intervals. For hedonic food intake measurements, in addition to chow mice had access to chocolate (“Milka Naps Vollmilch”, 5.3 kcal/g, Mondelez, Bremen, Germany) either during the whole day (hedonic profiling) or during 2 h at night (ZT16-18) or day (ZT4-6) (snacking). Chow/chocolate consumption was determined by weighing before and after snack access. **Overconsumption** was determined by dividing total consumption under choice conditions by chow or water consumption under non-choice conditions as previously described (total caloric intake [chow + chocolate]/homeostatic ad libitum chow caloric intake) [39].

### 2.3 Homeostatic and hedonic drinking behavior

To determine liquid intake rhythms, mice were individually placed in lickometer cages (Model 80380, Campden Instruments, Loughborough, UK) with three bottles of tap water for two days under standard LD conditions. After this time, two bottles of comparable intake were chosen and water in the less preferred one was replaced by 10-% sucrose solution (w/v in water). **Liquid intake** (measured as number of licks) was registered using Scurry Activity Monitoring Software (Model 86165, Campden Instruments) every 6 h. **Sucrose preference** was calculated as the percentage of licks registered for the 10-% sucrose solution bottle. **Overconsumption** was calculated as described for food intake measuring licks instead of calories.

### 2.4 Body composition analysis

For nuclear magnetic resonance (NMR) measurements, 12-week-old mice were placed in a Bruker Minispec (Bruker, Billerica, USA). Data were analyzed with Minispec Plus software (Bruker) following the manufacturer's instructions.

### 2.5 Temperature measurements

Basal body, brown adipose tissue (BAT) and inner ear temperature were assessed at different timepoints using a thermal image camera (T335, FLIR Systems, Wilsonville, USA). Infrared pictures were processed and analyzed with FLIR Tools (FLIR Systems) [43].

### 2.6 Hormone profile

Blood samples were collected every 4 h from mixed-sex mice aged 2–3 months. Serum leptin levels were measured according to the manufacturer's instructions using the Mouse Leptin ELISA Kit (Crystal Chem Inc.).

### 2.7 Activity monitoring

Locomotor activity of singly housed mice was recorded in transparent plastic cages (#1155M, Tecniplast, Buguggiate, Italy) equipped with a running-wheel (#6083, Trixie, Tarp, Germany) and analyzed in 5-min bins with ClockLab Activity Analysis software (Actimetrics, Evanston, USA) as described [44]. Mice were kept for 2 weeks under LD conditions, followed by 2 weeks of constant darkness (DD) and two weeks of constant light (ca. 100 lux, LL) to monitor entrained and free-running circadian locomotor rhythms.

### 2.8 Indirect calorimetry

An open-circuit indirect calorimetry system (PhenoMaster, TSE Systems, Bad Homburg, Germany) was used to measure oxygen consumption, carbon dioxide production, respiratory quotient and energy expenditure as described [45]. Mice were kept in the calorimetry system for 3–4 days after being adapted to the specific drinking bottles. Cage temperature, food and water intake were also measured. Only the last 2 days were used for analysis.

### 2.9 Leptin treatment

Mice were anaesthetized with isoflurane and fixed in a stereotact (Model 1900; Kopf Instruments, Tujunga, USA). Coordinates for the lateral ventricle were x = 1.1 mm, y = 0.1 mm and z = 2.2 mm from *bregma*. One hole was drilled for the cannula and two more for the screws. After insertion of the cannula, the mice were allowed to recover for one week. Before injections, mice were fasted for 24 h. 4 μg leptin (recombinant mouse, *E. coli* derived, R&D Systems, Minneapolis, USA) in 2 μl artificial cerebrospinal fluid (aCSF) or 2 μl aCSF (as control) were injected. 20 min after the injection, mice were anaesthetized with sodium pentobarbital (*i.p.*, 160 mg/ml, 10 μl/g bodyweight) and perfused as described [39]. Brains were dissected and stored for 4–16 h in 4 % paraformaldehyde (PFA) at 4 °C. PFA was exchanged with 30 % sucrose solution and specimens stored at 4 °C until the brains were sunken to the bottom of the tube. Brains were frozen in isopentane/dry ice and stored at −80 °C. Sectioning was performed in a cryostat (CM3050S, Leica Biosystems, Nussloch, Germany) at 35 μm. Sections were stored free-floating in cryoprotectant at −20 °C.

### 2.10 Real-time quantitative PCR (qPCR)

Brains were isolated after decapitation at the indicated time points, frozen on dry ice and cryo-sectioned at 12 μm. Sections containing the area of interest (arcuate nucleus, ARC; VTA or SCN) were placed on membrane slides (1.0 PEN, Carl Zeiss Microimaging, Oberkochen, Germany) and trimmed by laser dissection under a microscope (Palm Microbeam, Carl Zeiss Microimaging). For each mouse, three sections were pooled. RNA isolation was conducted using Direct-zol RNA Micro-Prep Kit (Zymo Research, Irvine, USA) following the manufacturer's instructions. High-capacity cDNA Reverse Transcription Kit with random hexamer primers (Life Technologies, Carlsbad, USA) was used for reverse transcription. qPCR was performed on a CFX96 thermocycler (Bio-Rad, Munich, Germany) running Bio-Rad CFX Manager 3.0 and GoTaq qPCR Master Mix (Promega, Madison, USA). *Eef1α* was used as reference gene, and the ΔΔC_t_-method [46] was used to calculate relative mRNA expression levels. Primer sequences were: *Bmal1*: 5′-CCTAATTCTCAGGGCAGCAGAT-3′ and 5′-TCCAGTCTTGGCATCAATGAGT-3’; *Eef1α*: 5′-TGCCCCAGGACACAGAGACTTCA-3‘ and 5’-AATTCACCAACACCAGCAGCAA-3’; *Dbp*: 5′- AATGACCTTTGAACCTGATCCCGCT-3′ and 5′-GCTCCAGTACTTCTCATCCTTCTGT-3’; *ObRb*: 5′- AATGACGCAGGGCTGTATGT-3′ and 5′- TCAGGCTCCAGAAGAAGAGG-3′, *Nr1d1* 5′*-AGCTCAACTCCCTGGCACTTAC-*3′ and 5′*-CTTCTCGGAATGCATGTTGTTC-*3’*;* and *Per1 5′-AGTTCCTGACCAAGCCTCGTTAG-3′,* and *5′-CCTGCCCTCTGCTTGTCATC-3’.*

### 2.11 Immunohistochemistry (IHC)

Cryo-sections were used for phospho-STAT3 IHC with phospho-STAT3 (Tyr705) rabbit mAb (dilution of 1:250/c-FOS rabbit monoclonal antibody (9F6) LOT: 12, Cell Signaling Technology, Massachusetts, USA.) in a free-floating setup. Sections were transferred with a brush into 12-well plates and washed 6 × 5 min with phosphate buffer (PB), followed by a 20-min incubation with 1 % NaOH and 1 % H_2_O_2_. If not stated differently, all steps were conducted at room temperature with gentle shaking. Next, sections were washed, immersed with 0.3 % glycine in PB for 10 min, washed again and incubated in 0.03 % SDS in PB for 10 min. Sections were blocked with 0.5 % PB-Triton X and 5 % normal goat serum (Cell Signaling Technology) for 1–2 h. Incubation with the primary antibody (1:200 dilution, in blocking solution) occurred overnight at 4 °C. The next day, sections were washed and incubated with the secondary antibody (1:600, goat anti-rabbit IgG biotinylated, Dako, Agilent, Santa Clara, USA) for 1 h. This was followed by washing and an incubation with ABC solution (Vectastain Elite ABC Kit for rabbit IgG, Vector Laboratories, Maravi Life Sciences, Burlingame, USA) for 2 h. Diaminobenzidine solution (DAB Peroxidase Substrate Kit, Vector Laboratories) was added for 3–5 min and then washed with PB. Sections were transferred with a brush onto gelatine-coated slides. Sections were dehydrated with increasing ethanol concentrations followed by xylene and mounted with DPX (Sigma–Aldrich, St. Louis, USA). Cells on micro images were counted by a condition-blind person with Fiji (ImageJ, NIH, Bethesda, USA) [47]. The c-FOS experiment was conducted on *ObRb.Cre* and *ObRb.Bmal1* mice. Both genotypes were offered a snack for 20 min, after which the animals were immediately sacrificed. c-FOS induction was then compared to that of animals that did not receive snack treatment, allowing us to calculate the fold induction.

### 2.12 Adipocyte histology

Adipocytes sections were prepared according to our previously published procedures [48]. After isolation, epididymal WAT samples fixed with 4% paraformaldehyde/PBS overnight. In brief, WAT samples were dehydrated with alcohol and embedded in paraffin. Afterwards, we prepared 6-μm sections with a microtome, and we stained the sections with hematoxylin-eosin. Section images were used to determine adipocyte sizes using Image J software (National Institutes of Health, Bethesda, MD).

### 2.13 Software and statistics

Locomotor activity period was determined by χ^2^ periodogram analysis with ClockLab (Actimetrics). Diurnal rhythms of gene expression and metabolic data such as food intake, water intake, sucrose preference and overconsumption were assessed for each individual across the day with CircaCompare [49] with a p-value cut-off of 0.05. CircaCompare indicates that a data set is rhythmic by fitting data to a cosine curve. CircaCompare was used to assess whether the dataset exhibited circadian rhythmicity and to evaluate changes in estimated rhythm parameters, with a particular focus on amplitude. For the data involving repeated measurements from the same individual, we used circa_single_mix in R studio. All other statistical analyses were performed using GraphPad Prism Version 8 or 9 (GraphPad, La Jolla, USA). Data were tested for normal distribution using the Shapiro–Wilk test for low-replicate (n = 3–5) and Kolmogorov–Smirnov test for larger data sets (n = 6 or more). In cases where the data did not follow a normal distribution, they were log10 transformed, and parametric tests were used for subsequent analyses. Multiple comparisons were conducted using a two-way ANOVA (or two-way repeated measures ANOVA when the analysis included time variables) or mixed-model analysis for data sets with missing values. Sidak post-tests were used for time series analyses. Two-group comparisons were performed using Student's t-tests. Specific tests are indicated in the figure legends and in Table S1.

## 3 Results

### 3.1 Altered diurnal food and liquid intake rhythms in leptin deficient mice

**Figure 1:**
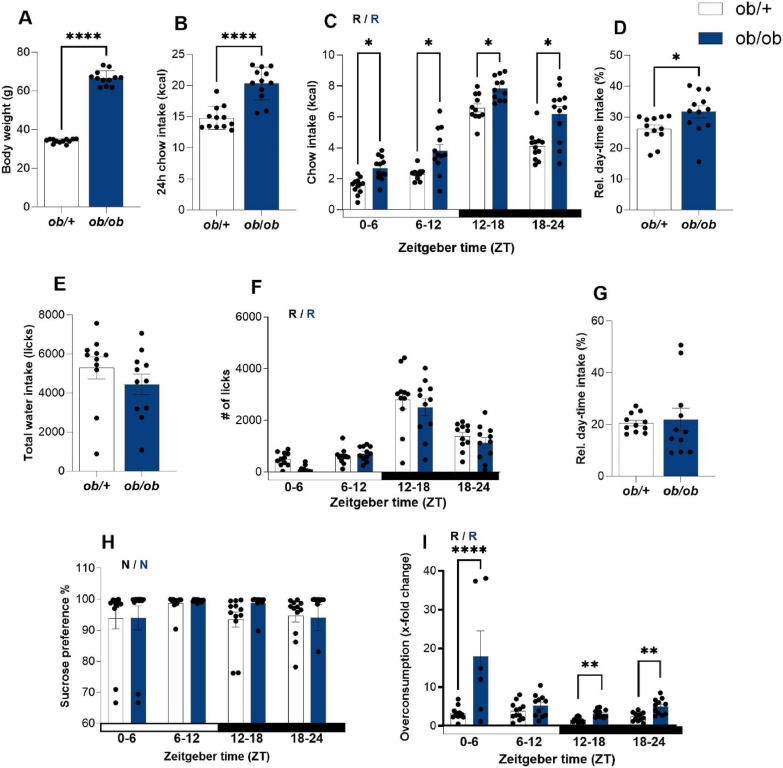
**Exaggerated rhythms in hedonically driven overconsumption in leptin deficient mice (*ob/ob* mice).** All data (mean +/− SEM) from *ob/ob* mice (blue) and controls (*ob/+*, white). **(A)** Body weight development over 16 weeks (n = 12). **(B)** 24-hour food consumption (n = 12). **(C)** Comparative daily profile of daily food intake (n = 12). **(D)** Relative light phase intake (n = 12). **(E)** 24-hour water intake (n = 12). **(F)** Comparative daily profile of daily water licks (n = 12). **(G)** x-fold water consumption per night compared to total day-time intake (n = 12). **(H)** Sucrose preference (water vs. sucrose + water, n = 11–12). **(I)** Overconsumption (n = 6–12). R and N indicate whether the dataset is rhythmic (p < 0.05) or non-rhythmic (p > 0.05). T-test, 2-way RM -ANOVA or Mixed-effect model with Sidak's multiple comparation test and CircaCompare rhythmicity analysis and Circa_single_mixed rhythmicity analysis in the case of repeated measurement analysis. ∗*p* < 0.05, ∗∗*p* < 0.01, ∗∗∗*p* < 0.001, ∗∗∗∗*p* < 0.0001.

To analyze the role of leptin signaling in the diurnal regulation of appetite, we studied food and liquid intake in leptin-deficient (*ob/ob*) mice. We hypothesized that if circulating leptin affects hedonic intake, *ob/ob* mice would exhibit altered hedonic intake (i.e., overconsumption), potentially disrupting their circadian rhythm of hedonic feeding. In line with previous reports, *ob/ob* mice showed increased body weight (Fig. 1A, Table S1) concomitant with elevated total food intake compared to heterozygous littermate controls (*ob/+*, Fig. 1B). In both genotypes, food intake over 24 h was rhythmic (Fig. 1C), with most of the chow being consumed during the dark phase. Notably, however, *ob/ob* mice consumed about a third of their food during the day, while in controls this proportion was slightly lower at around 25% (Fig. 1D). Water intake was comparable between genotypes regarding total intake (Fig. 1E), diurnal rhythmicity (Fig. 1F), and light/dark phase distribution (Fig. 1G). To test if hedonic aspects of liquid intake rhythms were similarly preserved, mice were given a choice of two bottles containing either water or a 10% sucrose solution. Under such choice conditions both genotypes strongly preferred sucrose over water with little variation over the course of the day (Fig. 1H). As previously shown for wild-type animals [39], overconsumption in comparison to no-choice conditions was rhythmic in control mice with peak overconsumption during the early light phase (Fig. 1I). This rhythm, though, was more exaggerated in *ob/ob* animals (Fig. 1I, amplitude difference *p* = 0.0006). Together, these data suggest that while leptin plays a tonic (i.e., time-independent) role in regulating homeostatic food intake, it may at the same time function as a phasic (i.e, time-of-day-dependent) modulator of hedonic appetite.

Importantly, these findings could also be influenced by the obesity stage in *ob/ob* mice rather than solely by the absence of circulating leptin. To further investigate this, we analysed overconsumption in wild-type mice fed a high-fat diet (HFD). After 10 weeks of HFD, mice were significantly heavier than normal chow (NC) fed animals (Fig. S1A), but HFD intake was rhythmic over the day (Fig. S1B). When HFD mice had the choice between HFD and chocolate, overconsumption was highest during the resting phase (Fig. S1C) but did not reach the same levels observed in leptin-deficient mice. These findings suggest that the phasic effect of leptin on overconsumption is not merely an effect of obesity.

Both, *ob/ob* and WT mice exhibited hedonic rhythms (rhythmicity: *ob/ob* p < 0.0001, *ob/+* p < 0.0001). However, *ob/ob* mice displayed altered rhythms, with overconsumption peaks being approximately five times higher than in controls. This suggests that the absence of circulating leptin does not eliminate hedonic circadian rhythms but rather exaggerates them. To identify the underlying mechanisms of this function, we examined whether the circadian clock plays a role in modulating overconsumption and the effects of leptin on homeostatic and hedonic centres of the brain in lean mice lacking a functional circadian clock.

### 3.2 Loss of circadian clock function in Per1/2 mutant mice alters leptin gating in ARC and VTA and blunts hedonic intake rhythms

**Figure 2:**
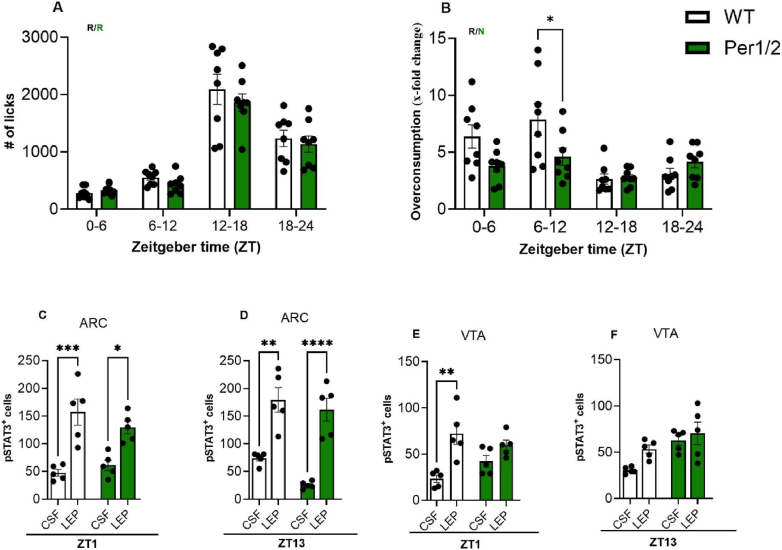
-**Loss of clock function (in *Per1/2* mice) alters leptin gating in the VTA and blunts hedonic intake rhythms.** All data (mean +/− SEM) from Per1/2 mice (green) and controls (wildtype, WT; white). **(A)** Total 24h water intake (n = 8). **(B)** Sucrose overconsumption (n = 8). **(C)** Preserved leptin gating in ARC at ZT1 (n = 5) and **(D)** at ZT13 (n = 5). **(E)** Altered leptin gating in VTA at ZT1 (n = 5) but no effect at **(F)** ZT13 (n = 5). R and N indicate whether the dataset is rhythmic (p < 0.05) or non-rhythmic (p > 0.05). 2-way ANOVA or 2-way RM ANOVA with Sidak's multiple comparation test and CircaCompare rhythmicity analysis Circa_single_mixed rhythmicity analysis. ∗*p* < 0.05, ∗∗*p* < 0.01, ∗∗∗*p* < 0.001, ∗∗∗∗*p* < 0.0001.

To investigate the general role of the circadian clock in regulating hedonic food intake, we used a clock deficient mouse model (*Per1/2* double knockouts) [40]. If leptin modulates hedonic appetite in a time-dependent manner, animals lacking a functional circadian clock should exhibit blunted overconsumption rhythms and a non-rhythmic sensitivity of leptin receptors to leptin stimulation in the brain. In the present study, we focused on reporting liquid intake rhythms and hedonic feeding behaviour in *Per1/2* knockout mice, as homeostatic feeding profiles have already been published previously [39]. Liquid intake over 24 h of both, *Per1/2* and control mice was rhythmic, as expected (Fig. 2A). However, the 24-hour hedonic overconsumption rhythm under choice conditions was blunted in *Per1/2* animals (amplitude difference *p* < 0.0001), with lower overconsumption during the second half of the light phase (ZT 6–12, Fig. 2B). Similar findings were observed in a different model of clock deficiency, where overconsumption amplitudes were markedly diminished (*Bmal1*-KO, Fig. S2, amplitude difference *p* < 0.0001) while no differences were observed for water-only intake. This effect was independent of general sucrose preference, which was comparable between clock-deficient and control mice (Fig. S3).

To investigate if central leptin sensitivity might be regulated by the circadian clock, fasted mice were *i.c.v.* (intracerebroventricularly) treated with leptin or CSF, and brain sections containing the ARC (involved in homeostatic appetite regulation) or the VTA (affecting hedonic appetite) were stained for pSTAT3 as a marker for activation of the leptin signaling cascade at two times of the day (ZT1 in the early morning and ZT13 in the early dark phase). Both genotypes showed increased pSTAT3 expression in the ARC after leptin treatment compared to CSF at both time points, indicating that the absence of the clock does not affect the leptin response in the ARC (Figure 2C and D). In the VTA, the effects of leptin were less pronounced than in the ARC, with significant induction observed only at ZT1 and a subtle, non-significant increase at ZT13 in wild-type animals (Figure 2E and F). However, clock-deficient mice did not exhibit a response to leptin at any time point. These results suggest a circadian gating of leptin signaling in the VTA, which is reflected in the blunted diurnal hedonic intake rhythms in clock-deficient mice.

### 3.3 The circadian clock regulates leptin receptor expression in appetite regulatory circuits and the SCN

**Figure 3:**
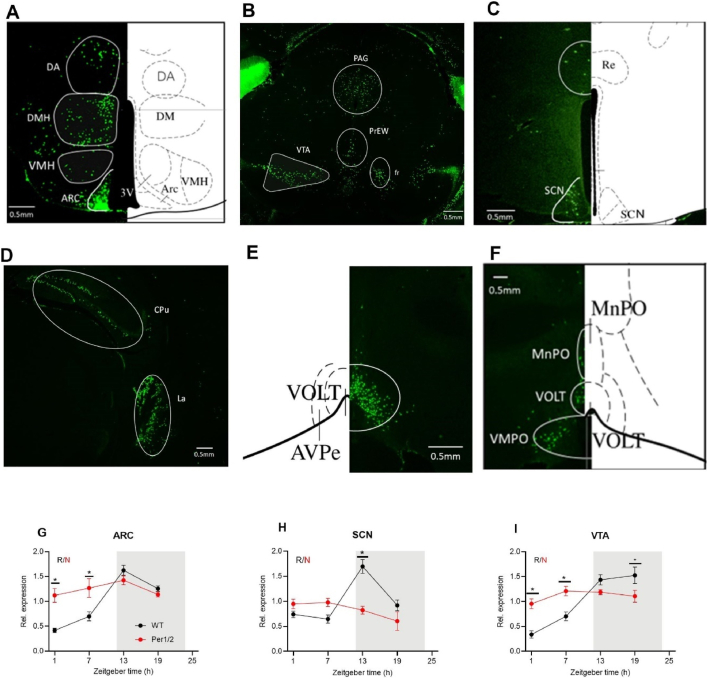
**The circadian clock regulates leptin receptor expression in the ARC (appetite control), the VTA (hedonic feeding/reward), and the SCN (circadian pacemaker).** All data (mean +/− SEM) from Per1/2 mice (red) and controls (wildtype, WT; black). **(A**–**F)***ObRb* expression mapping *ObRb.Bmal1*.Green mice. Microscopic images of *ObRb.Bmal1.*Green mice showing Cre recombination and therefore GFP expression in (**A**) nucleus arcuatus (interaural 2.10 mm, Bregma −1.70), (**B**) ventral tegmental area (Interaural 0.88 mm Bregma −2.92), (**C**) suprachiasmatic nucleus (interaural 3.34 mm, bregma −0.46 mm), (**D**) lateral amygdaloid nucleus (interaural 2.86 mm, bregma −0.94), (**E**) anteroventral periventricular nucleus (interaural 4.42 mm, bregma −0.62) and (**F**) ventromedial preoptic nucleus (interaural 4.42 mm, bregma −0.62). The overview of all represented areas within mouse brain (adapted from Allen Brain Atlas, brain explorer). (**G**) Blunted *ObRb* mRNA rhythms in Per1/2 mice in nucleus arcuatus (ARC, n = 4, qPCR assays), (**H**) suprachiasmatic nucleus (SCN, n = 4, qPCR assays) and (**I**) ventral tegmental area (VTA, n = 4, qPCR assays). R and N indicate whether the dataset is rhythmic (p < 0.05) or non-rhythmic (p > 0.05). 2-way ANOVA with Sidak's multiple comparation test and CircaCompare rhythmicity analysis. ∗*p* < 0.05, ∗∗*p* < 0.01, ∗∗∗*p* < 0.001, ∗∗∗∗*p* < 0.0001.

While changes in leptin levels themselves may provide a timing signal to central metabolic circuits [50], our data thus far were also in line with an alternative mechanism involving circadian regulation of leptin signaling at brain target sites. To assess this possibility, we analyzed expression of the long isoform of the leptin receptor, *ObRb* (*LepRb*), in the central nervous system. For this purpose, we crossed mice expressing CRE recombinase linked to the long isoform of *ObRb* (*ObRb-Cre*) with *ZsGreen* reporter mice and mapped fluorescence patterns throughout the brain. *ObRb-*driven recombination (and, thus, fluorescence) was detected in homeostatic appetite centers of the MBH, such as the ARC and the ventromedial nucleus of the hypothalamus (VMH; Fig. 3A). *ObRb-*positive cells were also found in areas involved in hedonic appetite regulation such as the VTA (Fig. 3B), and in the core circadian pacemaker, the SCN (Fig. 3C). In addition, fluorescence was found in further brain areas such as the lateral amygdaloid nucleus (La; Fig. 3D), which plays a role in processing sensory information corrected to anxiety, the anteroventral periventricular nucleus (AVPe; Fig. 3E), involved in the neuroendocrine control of ovulation in females, and the ventromedial preoptic nucleus (VMPO; Fig. 3F), which regulates sleep and body temperature.

In wild-type mice, mRNA levels of *ObRb* were cyclic over the day with a peak in the early night in the ARC and SCN and around midnight in the VTA (Figure 3G–I). To test the extent to which these rhythms were regulated by the clock gene machinery, we analyzed *ObRb* mRNA levels in *Per1/2* mutant mice. In the mutants, *ObRb* mRNA rhythms were blunted at high overall levels in the ARC (amplitude difference *p* < 0.0001), VTA (amplitude difference *p* < 0.0001) and the SCN (amplitude difference *p* < 0.0001; Figure 3G–I). Together, these data further support the idea that leptin reception in central appetite centers may be subject to circadian gating.

### 3.4 Circadian activity rhythms are preserved in ObRb.Bmal1 mice

**Figure 4:**
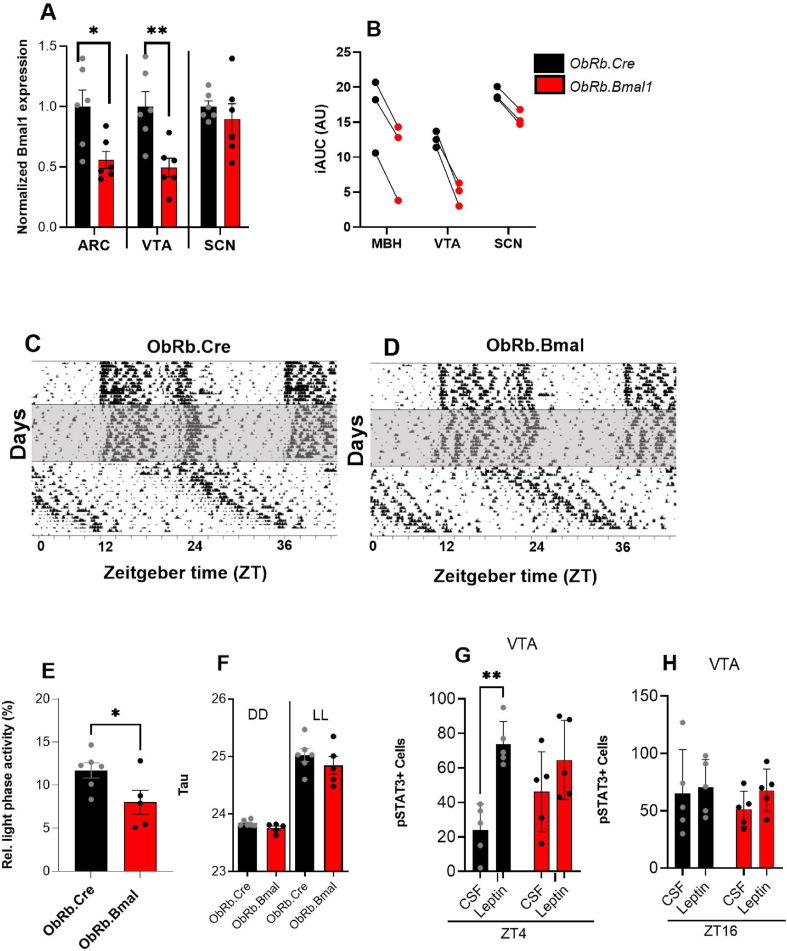
**Preserved circadian activity rhythms in *ObRb.Bmal1* mice.** All data (mean +/− SEM) from *ObRb.Bmal1* (red) and controls (*ObRb.Cre*, black). **(A)***Bmal1* expression in ARC (n = 6), VTA (n = 6) and SCN (n = 6). **(B**) A decrease in the area under the curve (AUC) for the expression of *Dbp*, *Per1*, and *Nr1d1* in the MBH, VTA and SCN. **(C)** Actograms of control and **(D)** mutant mice during LD, DD and LL condition. **(E)** Relative light phase running-wheel activity (n = 5–6). **(F)** Total activity during the dark phase (DD, active phase; n = 5–6) and light phase (LL, inactive phase; n = 5–6). **(G)** Increased response in the VTA after the leptin treatment in control mice at ZT4 but **(H)** no effect at ZT16. T-test, 2-way ANOVA with Sidak's multiple comparation test. ∗*p* < 0.05, ∗∗*p* < 0.01, ∗∗∗*p* < 0.001, ∗∗∗∗*p* < 0.0001.

Our findings provided evidence that the circadian clock may regulate leptin receptors in the VTA to modulate hedonic food intake. However, as both *ob/ob* and *Per1/2* KO models involve global gene mutations, potential systemic effects could influence the results. To address this limitation, we generated a mouse model with clock ablation specifically in ObR-expressing neurons (*ObRb.Bmal1.*KO), thereby abrogating clock function in these cells. Recombination was validated by qPCR for *Bmal1* on laser-dissected ARC, VTA, and SCN tissue. Of note, in none of these tissues *Bmal1* deletion was complete with *ObRb.Bmal1* mice exhibiting approximately 50% lower *Bmal1* mRNA levels in the ARC and the VTA while no significant reduction of *Bmal1* mRNA was observed in the SCN compared to controls (*ObRb.Cre*; Fig. 4A).

To confirm the effectiveness of *Bmal1* recombination in disrupting clock function in these tissues, we measured the daily mRNA expression profiles of the clock-controlled genes, *Dbp*, *Per1*, and *Nr1d1,* in both genotypes (Fig. S4). Consistent with the observed partial suppression of *Bmal1* expression in *ObRb.Bmal1* mice, the daily dynamics of *Dbp*, *Per1*, and *Nr1d1* activation were consistently dampened in the MBH and VTA (amplitude measured with CircaCompare and the reduction confirmed with AUCs of gene expression profiles, Fig. 4B and Fig. S4). Following this observation, we confirmed that the circadian clock remained unaffected by analysing physical activity patterns. Locomotor activity patterns under different light conditions (12-hour light/12-hour dark – LD, constant dark–DD, and constant light–LL) were comparable between both genotypes (Figure 4C and D). However, *ObRb.Bmal1* mice exhibited slightly less relative light (i.e., rest) phase activity under LD conditions (Figure 4E–F). Like the test performed in *Per1/2* (Figure 2E, F), we tested leptin sensitivity in control and *ObRb.Bmal1* mice by *i.c.v* treatment with leptin or CSF. VTA was stained for pSTAT3 as a marker for activation of the leptin signaling cascade after treatment at two times of the day (ZT4 in the early morning and ZT16 in the early dark phase). At ZT4, the control group showed increased pSTAT3 expression after the leptin treatment while at ZT16 none of the genotypes showed significant responses (Figure 4G and H).

In sum, our data show that *ObRb*-driven deletion of *Bmal1* may alter clock function in the ARC and VTA, while SCN pacemaker function and locomotor activity rhythms are preserved in these mice.

### 3.5 Energy metabolism in *ObRb.Bmal1* mice

**Figure 5:**
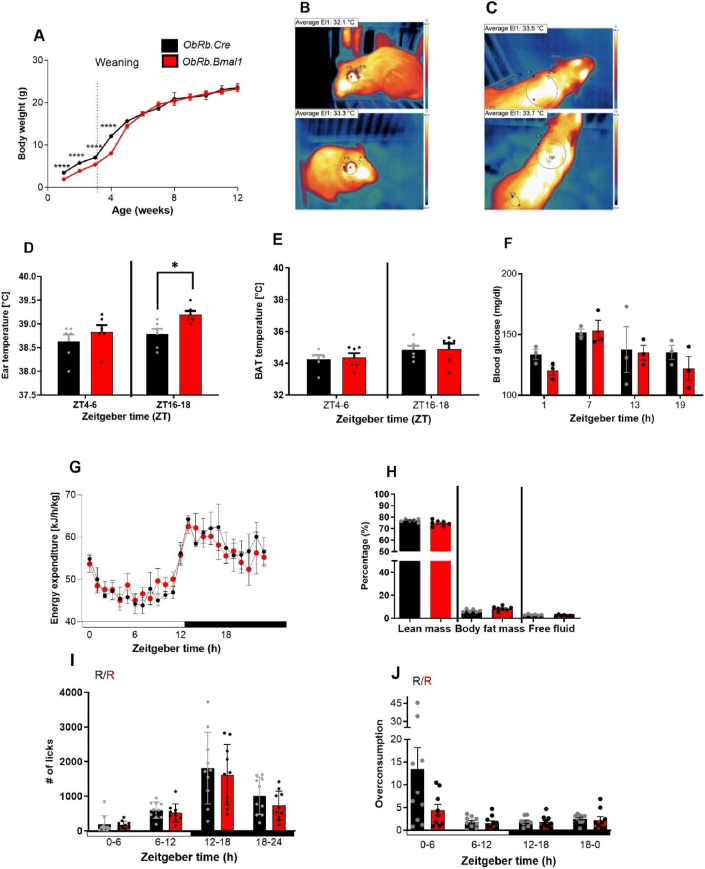
***Bmal1* knock-out in ObRb-expressing neurons does not alter baseline metabolic characteristics.** All data (mean +/− SEM) from male *ObRb*.*Bmal1* (red) and *ObRb.Cre* mice (black) as controls. **(A)** Body weight development over 12 weeks (n = 16–22). **(B)** Infrared ear temperature detection. **(C)** Infrared brown adipose tissue (BAT) temperature detection (left *ObRb.Cre*, right *ObRb.Bmal1*). **(D)** Comparison of inner-ear temperature at ZT4-6 (n = 6) and ZT16-18 (n = 6). **(E)** Comparison of BAT temperature at ZT4-6 (n = 6) and ZT16-18 (n = 6). **(F)** Rhythmic blood glucose at different time points (n-3). **(G)** Energy expenditure over 24h in both genotypes during LD conditions (n = 4). **(H)** Comparation of percentage of lean mass, body fat mass and free fluid (n = 6–7). **(I)** Water consumption over 24 h (n = 10). **(J)** Blunted sucrose overconsumption in the active phase in *ObRb.Bmal1* mice (n = 10). R and N indicate whether the dataset is rhythmic (p < 0.05) or non-rhythmic (p > 0.05). T-test, 2-way RM ANOVA or mixed-effect analysis with Sidak's multiple comparation test and Circa_single_mixed rhythmicity analysis. ∗p < 0.05, ∗∗p < 0.01, ∗∗∗p < 0.001.

Considering the observed effect of *ObRb*-driven *Bmal1* deletion on circadian clock function in metabolically relevant brain regions, we next analyzed energy metabolism under standard diet conditions in these animals. *ObRb.Bmal1* mice exhibited slightly lower body weight during lactation and gained weight significantly slower during the first week after weaning, but they caught up soon after that (Fig. 5A). At 12 weeks of age, daily chow intake rhythms in *ObRb.Bmal1* mice were comparable to those of controls, and overall food intake was similar (Fig. S5). The circulating leptin displayed a robust circadian rhythm with no significant differences between genotypes (Fig. S6). Consistently, inner-ear temperature (Fig. 5B, used as a proxy for core body temperature) and interscapular skin temperature (Fig. 5C an indicator of brown adipose tissue (BAT) activity) did not show marked differences between genotypes (Figure 5D–E). Daily profiles of blood glucose levels and energy expenditure were preserved in *ObRb.Bmal1* mice (Figure 5F, G). At 12 weeks of age, body composition was similar between genotypes, and non-significant variation in fat mass, lean mass, and free body fluid were observed (Fig 5H). At the same time, rhythmic expression of leptin receptor mRNA was lost in *ObRb.Bmal1* KO mice in both the SCN and VTA, while in the ARC rhythmicity was reduced (Fig. S7 A-C). In summary, loss of *Bmal1* in ObRb + neurons affected leptin receptor expression in metabolically relevant brain areas but had little effect on baseline metabolic characteristics.

### 3.6 Blunted hedonic appetite rhythms in ObRb.Bmal1 mice

We hypothesized that if the circadian clock in *ObRb*-expressing neurons plays a role in regulating hedonic feeding behaviour, we should observe a disruption in the hedonic intake in *ObRb.Bmal1* KO mice in a time-of-day dependent manner. To compare homeostatic and non-homeostatic appetite regulation between *ObRb.Bmal1* and control mice, we analyzed drinking behavior under choice and no-choice conditions. Daily water consumption rhythms of *ObRb.Bmal1* mice and controls were similar with peaks in the early dark phase (Fig. 5I). Under choice conditions, sucrose solution preference was comparable between genotypes with little variation over the course of the day (Fig. S8). However, hedonic overconsumption rhythms of sucrose solution were blunted in *ObRb.Bmal1* mice with (2-3-fold, from 5.93 to 1.34, amplitude difference p = 0.022) reduced overconsumption during the early light phase (ZT0-6) (Fig. 5J).

**Figure 6:**
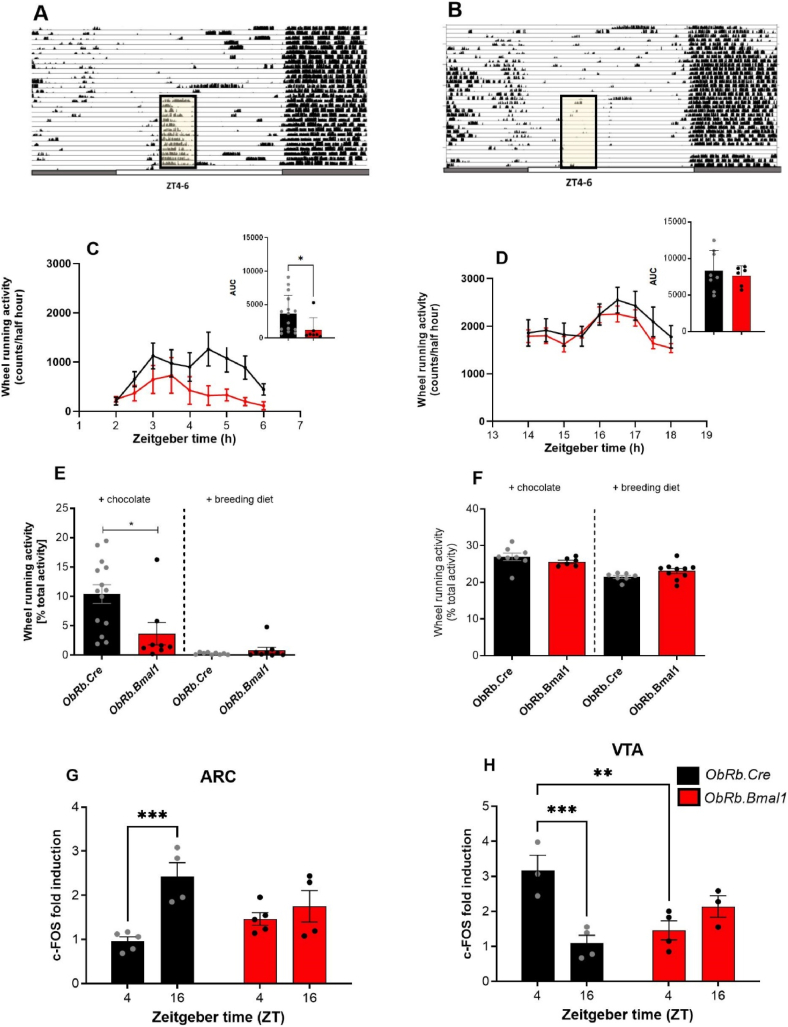
**Hedonic appetite regulation rhythms are dampened in *ObRb.Bmal1* mice.** All data (mean +/− SEM) from Male *ObRb*.*Bmal1* (red) and *ObRb.Cre* mice (black) as controls. **(A)** Actograms of control and **(B)***ObRb.Bmal1* during hedonic snack at ZT 4–6. **(C)** Wheel running activity during hedonic snack at ZT 4–6 and area under the curve (n = 8–14). **(D)** Wheel running activity during hedonic snack at ZT16-18 and area under the curve (n = 6–8). **(E)** Average wheel running activity during chocolate intake and breeding diet (n = 9) at ZT 4–6 and ZT 16–18 (**F,** n = 6–10). **(G)** More c–FOS–positive cells in the morning than in the evening snack-time in ARC in control mice (n = 5–4). **(H)** chocolate mediates neuronal activity induction measure via c-FOS staining in the morning snack-time and evening snack-time in VTA (n = 3–4). T-test and 2-way ANOVA with Sidak's multiple comparation test. ∗p < 0.05, ∗∗p < 0.01, ∗∗∗p < 0.001.

To further corroborate this finding, we tested a different paradigm for investigating the effects of a hedonic diet. Mice were repeatedly given a palatable snack (chocolate) or a less palatable food item (breeding diet) either in the light phase (ZT 4–6) or in the dark phase (ZT 16–18) [51]. After two weeks, wheel-running activity was compared as a sign of anticipation of the snack (Figure 6A–D). In the morning snack group, chocolate-fed *ObRb.Cre* mice showed a marked increase in running-wheel activity compared to chocolate-fed *ObRb.Bmal1* mice starting at around 2 h before snack access and becoming significant during the time of snack access (Figure 6B and C). In control mice, this response of chocolate-fed mice compared to breeding diet-fed mice was much more pronounced than in *ObRb.Bmal1* mice, indicating less hedonic effect in the mutants (Fig. 6E). When the snack was given in the evening, the increase in activity was much less prominent and a lesser effect between snack types or genotypes were observed (Figure 6D, F).

After the 20-min snack access, animals were immediately sacrificed and brain sections containing the ARC and the VTA stained for c-FOS expression as marker of neuronal activation. FOS induction was compared to that of animals that did not receive snack treatment. In the ARC, chocolate-induced c-FOS expression was time-of-day dependent in control, but not in *ObRb.Bmal1* mice (Fig. 6G). In the VTA, however, c-FOS induction in controls was higher in the morning, while in mutant mice, time-of-day differences were no longer significant with lower induction during the day and higher induction during the night compared to control animals (Fig. 6H). Together, these data support the idea that circadian clocks in ObRb + neurons affect hedonic appetite rhythms and neuronal activation in the VTA.

### 3.7 Decreased susceptibility to early high-fat diet in ObRb.Bmal1 mice

**Figure 7:**
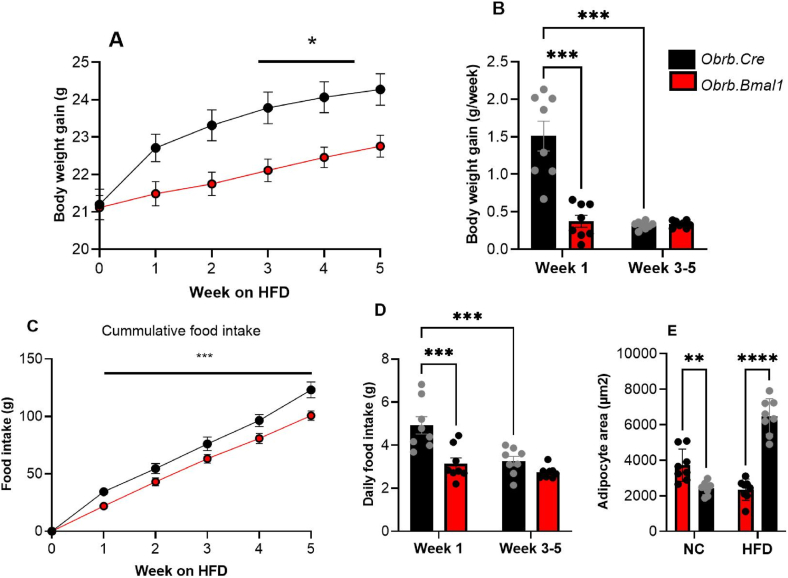
**Decreased susceptibility to early high-fat diet in *Obrb.Bmal1* mice.** All data (mean +/− SEM) from male *ObRb*.*Bmal1* (red) and *ObRb.Cre* mice (black) as controls. **(A)** Body weight development over 5 weeks under high-fat diet (HFD, n = 8). **(B)** Averaged body weight gain during week 1 and weeks 3–5 (n = 8). **(C)** Cumulative food intake over 5 weeks of HFD (n = 8). **(D)** Averaged daily food intake during week 1 and weeks 3–5 (n = 8). **(E)** Adipose hypertrophy under obesogenic diet conditions. 2-way ANOVA and 2-way RM ANOVA with Sidak's multiple comparation test. ∗p < 0.05, ∗∗p < 0.01, ∗∗∗p < 0.001.

Our data so far indicated reduced sensitivity to hedonic overconsumption in *ObRb.Bmal1* mice. We therefore hypothesized, that under palatable diet conditions they should be more protected from caloric overconsumption. To test this, we fed *ObRb.Bmal1* and control mice with a 60% HFD for 5 weeks. During this time, both genotypes increased their body mass. However, *ObRb.Bmal1* mice showed a significantly lower body weight compared to control mice starting in week 1 (Fig. 7A). This effect was mostly due to a rapid weight gain in the control mice in week 1 on HFD which was not observed in *ObRb.Bmal1* mice (Fig. 7B). Reduced weight gain in the mutants was accompanied by lower overall food intake compared controls in the first days following the introduction of the HFD (Figure 7C and D). At the end of the experiment, increased overall lower intake resulted in less adipose hypertrophy in *ObRb.Bmal1* compared to control mice (Fig. 7E). These results suggest that loss of clock function in *ObRb*-expressing neurons protects mice from the anabolic effects of palatable diets during the first phase of exposure.

## 4 Discussion

Obesity is a widespread phenomenon of pandemic proportions, primarily attributed to physical inactivity and the consumption of high-calorie, palatable foods typical of Western diets [52]. The adipose tissue-derived hormone leptin is a key regulator of appetite. However, in obese individuals, despite elevated plasma leptin levels, its satiation effect is markedly diminished [53]. Leptin controls homeostatic appetite and energy expenditure primarily by acting on AgRP and POMC neurons in the arcuate nucleus (ARC) of the mediobasal hypothalamus [12]. The homeostatic feeding rhythms depend on circadian clocks in AgRP neurons which integrate hormonal input from the periphery [54,55]. In addition to its role in homeostatic feeding, leptin can also modulate hedonic appetite [56]. In VTA dopaminergic neurons, leptin activates JAK-STAT signaling, leading to in phosphorylation of STAT3 and a reduction in dopaminergic tone, thereby decreasing hedonic hunger [12]. At the same time, circadian clocks in VTA dopaminergic neurons further regulate circadian rhythms in the susceptibility to hedonic overconsumption [39].

### 4.1 Changes in hedonic, but not homeostatic feeding rhythms in *ob/ob* mice

Despite their incapacity to produce leptin, *ob/ob* mice retain both their daily homeostatic feeding and hedonic overconsumption rhythms. Moreover, mutant mice show exaggerated hedonic intake rhythms, particularly during the resting phase—a critical period, as studies indicate that eating during this phase increases susceptibility to uncontrolled eating and obesity [57]. The absence of circulating leptin in this mouse strain leads to more overconsumption while retaining its circadian variation, indicating that circadian variations in leptin levels do not directly control hedonic overconsumption rhythms. Blunted rhythmic hedonic overfeeding behavior in clock-deficient mutant mice in the present study (*Per1/2, Bmal1*-KO mice) and others (*Per1* and *Per2* single mutants) provides further evidence that hedonic food intake dependents on a circadian mechanism [39,58]. The fact that circulating leptin is not the sole regulator of hedonic overconsumption rhythms led us to investigate whether the diurnal variation of leptin receptors in the brain's hedonic centres could be involved in the exaggerated hedonic intake rhythms. In our study, altered sucrose intake in animals lacking clock genes was accompanied by changes in leptin gating in ARC and VTA, the homeostatic and hedonic centers of the brain. The VTA, known for affecting reward consumption shows daily rhythms in firing rates [59], which could contribute to the daily variation in the vulnerability to hedonic stimuli [39,58].

### 4.2 The role of circadian clocks in modulating hedonic feeding

Our findings indicate that changes in leptin secretion do not drive hedonic feeding. An alternative scenario could involve the temporal modulation of leptin signaling at the level of leptin receptive neurons, i.e., circadian leptin gating. However, since both *ob/ob* and *Per1/2* KO models involve global knockouts, potential systemic effects could influence the results. To address this limitation, we generated a mouse model with circadian clock ablation specifically in ObRb-expressing neurons (*ObRb.Bmal1 KO*). *ObRb* mediates leptin signaling, and its expression is not only limited to those brain regions that control hunger, but we also found ObRb in La, AVPe or VMPO, suggesting that it could be involved in diverse neuronal processes. Notably, leptin receptors in the VTA are of particular interest as this region is part of the dopaminergic system, which influences the consumption of drugs and palatable foods and drinks [60,61]. These findings indicate that leptin signaling in the brain is not only involved in suppressing nutrient intake but also acts on motivation and the reward system [62].

### 4.3 Altered body weight development but normal adult metabolic function in ObRb.Bmal1 mice

The baseline metabolic characteristics of *ObRb.Bmal1* mice including body weight development, homeostatic food intake, RER, and energy expenditure were largely comparable to those of controls (*ObRb.Cre*). This suggests that the genetic ablation of *Bmal1* in leptin receptor expressing neurons does not significantly affect these general traits or overall brain development. Circadian fluctuations in circulating leptin levels are known to be disrupted in global clock-deficient mouse models, including *Bmal1* [63], *Per1/Per2*, and *Cry1/Cry2* knockouts [33]. However, these alterations were not observed in *ObRb.Bmal1* knockout mice compared to controls, suggesting that selective clock ablation in ObR-expressing neurons does not significantly impact the circadian rhythm of leptin secretion. Although homeostatic feeding and drinking behaviors remained rhythmic and unaffected, hedonic overconsumptive behavior under choice conditions was diminished, particularly at the start of the inactive phase when overconsumption was highest in control mice [39]. Its widely known that a high-fat diet is a risk for overconsumption due to is palatable effect [64]. Studies conducted with rodents have shown that introduction of a hedonic diet such as high-sugar or high-fat diet impacts feeding behavior [65]. Eating a novel food item can increase DA release in VTA target regions such as the medial prefrontal cortex and nucleus accumbens increasing the excitement for the new food [66]. The deletion of leptin receptors in *ObRb.Bmal1* mice may have also reduced their initial susceptibility to diet-induced obesity by altering receptor activity in the intestine, potentially affecting nutrient absorption [67]. Our results indicate that overconsumption of a high-fat diet is significantly elevated when this palatable diet is first introduced to *ObRb.Cre* mice (control) compared to *ObRb.Bmal1* mice. Specifically, in the first week *ObRb.Bmal1* mice consumed 35 % less HFD and gained 25 % less weight than control mice. Additionally, control mice exhibited greater adipose hypertrophy compared to *ObRb.Bmal1* mice. These results indicate that clocks in leptin-receptive neurons play a role in controlling the hedonic response to a new diet. Other studies have found that males are more susceptive to overeating during the introduction of a novel food [65]. Further investigations are needed to assess the susceptibility of both female and male *ObRb.Bmal1* mice to a hedonic diet.

Introducing a new diet can impact the animal's feeding behavior during early development. After birth, mammals rely entirely on maternal milk to obtain nutrients, but soon they incorporate a variety of food types. The weaning period appears to be a susceptible moment for control mice (*ObRb.Cre*) since they had a higher increase in body mass compared with *ObRb.Bmal1* pups suggesting that the deletion of leptin receptor in the brain provides protection against higher energy consumption after switching to a solid diet. The impact of the introduction of a new diet during weaning has been reported in other studies [68,69]. For instance, whereas leptin receptor-deficient pups reduce their growth in the present study, leptin-deficient pups (*ob/ob*) increased their body mass compared to *ob/+* controls specially under high-fat diet [68]. In another study, *Clock* mutant and control pups show similar growth rates during their first 5 weeks of life but after weaning, *Clock* mutant pups are heavier contrasting with our results [69]. Further investigations are necessary to assess if deletion of clock function specifically in leptin receptor-positive neurons leads also to an protection against overconsumption under highly palatable diets such as high-fat or high-sugar diet.

### 4.4 Altered hedonic feeding rhythms and novelty-associated vulnerability to HFD in *ObRb.Bmal1* mice

Our study demonstrates that abolishing circadian clock function in leptin receptor-expressing neurons alters circadian rhythms in ARC and VTA while leaving the master clock and locomotor activity rhythms intact. This is exemplified by the reduction of *Bmal1* expression in ARC and VTA, but not in the SCN. Additionally, the *ObRb.Bmal1* mice preserved their locomotor activity when placed in either constant darkness or constant light conditions. However, wheel-running activity was significantly higher during the chocolate snack consumption in the daytime in *ObRb.Cre* compared to *ObRb.Bmal1* animals, suggesting a circadian influence on leptin receptor sensitivity. During the nighttime, snacking breading diet affected wheel-running activity slightly less compared to chocolate.

Although the number of c-Fos positive cells in the ARC was similar between both genotypes (*ObRb.Cre* and *ObRb.Bmal1*), the timing of c-Fos activation in response to snacking differed. Neuronal activity in ARC was significantly higher during the nighttime compared to the daytime, indicating increased sensitivity of the leptin receptors over the day. The leptin receptors have been found previously in the VTA and other hypothalamic and extrahypothalamic sites [8,70]. Beyond time of day, other studies have found that the type of diet can affect ARC neuronal activity, reporting a reduction in neuronal firing during high-fat diet compared to standard chow [71]. It is important to test different types of snacks, as the current study only examined chocolate, but other hedonic meals could lead to a different response. Moreover, chocolate snacking led to higher neuronal activation and increased wheel-running activity much more during the day compared to nighttime snacking. This effect was also observed following i.c.v leptin injection. Since there is a daytime-dependent difference in neuronal activation in the ARC, with no variation between *ObRb.Bmal1* and control mice, and considering the genotype-related differences in behavior, this suggests that the homeostatic appetite rhythm mediated by the ARC remains largely unaffected. Instead, this indicates that rhythmic regulation of hedonic appetite is influenced.

Previous findings further support this by revealing a peak in VTA neuronal activity at ZT3 [39]. Consistent with this, we observed increased response during hedonic consumption in control mice, whereas *ObRb.Bmal1* mice showed a reduction in this activity. This suggests that deletion of the core clock gene *Bmal1* in leptin receptor-expressing neurons reduces the vulnerability to overconsumption, highlighting the importance of the clock in *ObRb*-expressing neurons in regulating hedonic food intake. Notably, while the VTA is a key region in hedonic regulation, other brain areas expressing leptin receptors may also play a role in hedonic feeding behavior.

Homeostatic feeding originates from energy or metabolic deficits while hedonic feeding heavily involves environmental and psychological factors [12]. Our study demonstrates that, while overall feeding behavior remains unaffected, hedonic overeating may be specifically influenced by clock-dependent leptin receptor sensitivity. The mechanism by which the clock affects the leptin action via receptor control in the brain could serve as a model for how circadian clock regulates hormone actions and influence overconsumption and the risk of addiction. Understanding the circadian control of hedonic and homeostatic feeding is critical for developing medical recommendations for individuals with obesity or those at higher risk, including shift workers and individuals with circadian rhythm disorders. The present study focuses solely on leptin activity within the central reward system. However, further analysis is needed to examine the circadian regulation of other key hormone receptors in the VTA, such as ghrelin and insulin, which may also influence hedonic food intake behavior [20,72]. Moreover, the genetic manipulation in *ObRb.Bmal1* mice did not exclusively target specific brain regions. As a result, other tissues expressing leptin receptors, such as the intestine and intestinal immune cells [73,74], may also have been affected, potentially influencing some of the observed outcomes, particularly the ones relate to sugar or fat absorption [67,73,74].

## CRediT authorship contribution statement

**Jazmin Osorio-Mendoza:** Writing – review & editing, Writing – original draft, Validation, Investigation, Conceptualization. **Jana-Thabea Kiehn:** Writing – review & editing, Validation, Investigation, Conceptualization. **Sarah Stenger:** Writing – original draft, Validation. **Keno O. Heinen:** Validation, Investigation. **Laura Griewahn:** Validation, Investigation. **Christiane E. Koch:** Validation, Methodology, Investigation, Formal analysis, Conceptualization. **Undine Haferkamp:** Validation, Investigation. **Violetta Pilorz:** Writing – review & editing, Validation, Supervision, Investigation. **Johanna L. Barclay:** Validation, Investigation, Conceptualization. **Parth Joshi:** Writing – review & editing, Investigation. **Lisbeth Harder:** Writing – review & editing, Validation, Methodology, Investigation. **Olaf Jöhren:** Validation, Methodology, Investigation. **Peter Kühnen:** Writing – review & editing, Validation. **Gregor Eichele:** Writing – review & editing, Supervision, Methodology. **Henrik Oster:** Writing – review & editing, Writing – original draft, Resources, Funding acquisition, Formal analysis, Conceptualization.

## Declaration of competing interest

The authors declare that there are no competing interests with regard to the data and findings presented in this paper.

## Data Availability

Data will be made available on request.

## List of Abbreviations

aCSF: Artificial cerebrospinal fluid
AgRP: Agouti-related peptide
ARC: Nucleus arcuatus
AVPe: Anteroventral periventricular nucleus
BAT: Brown adipose tissue
BMAL1: Brain and muscle ARNT-like 1
CNS: Central nervous system
CLOCK: Circadian locomotor output cycles kaput
CRY: Cryptocrome
DD: Constant darkness
GFP: Green fluorescent protein
HFD: High fat diet
i.c.v.: Intracerebroventricularly
JAK: Janus kinase
LD: Light-dark conditions
MBH: Medio basal hypothalamus
mRNA: Messenger ribonucleic acid
NC: Normal chow
NMR: Nuclear magnetic resonance
NPY: Neuropeptide Y
Ob: Obese/leptin
ObRb: Obese receptor isotype b
PB: Phosphate buffer
PER: Period
PFA: Paraformaldehyde
POMC: Proopiomelanocortin
pSTAT3: Phosphorylated signal transducer and activator of transcription 3
qPCR: Quantitative polymerase chain reaction
SCN: Suprachiasmatic nucleus
VMH: Ventromedial hypothalamus
VMPO: ventromedial preoptic nucleus
VTA: Ventral tegmental area
WAT: White adipose tissue
WT: Wild-type
ZT: Zeitgeber time
