# Supplementary material for: Regulation of hedonic feeding rhythms by circadian clocks in leptin-receptive neurons
Source: Mol Metab. 2025 Jul 24;100:102221. doi: 10.1016/j.molmet.2025.102221 (PMC12356030; doi:10.1016/j.molmet.2025.102221)
Supplement: Supplementary data [file mmc1.docx]

**Supplements**


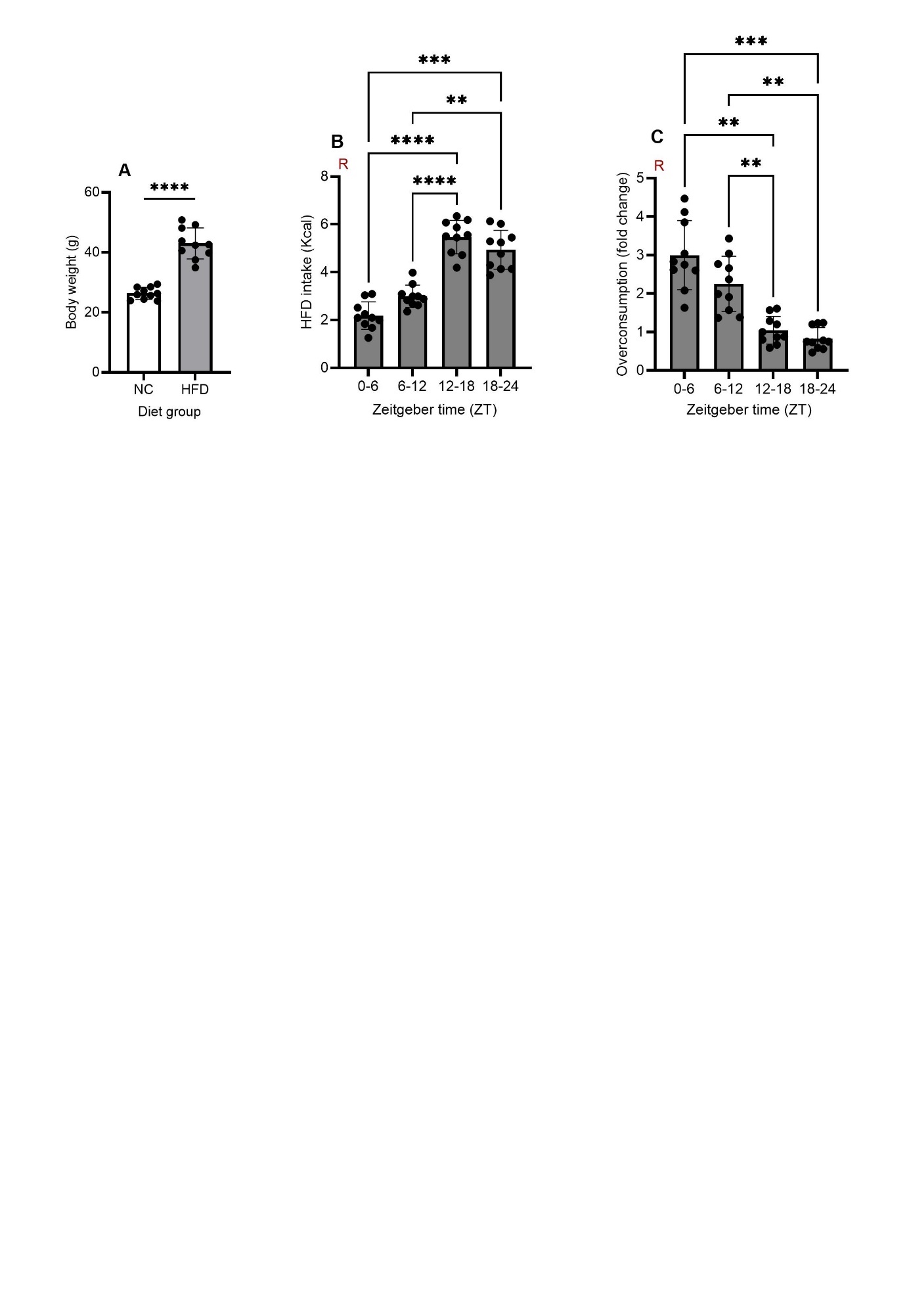


**Figure S1. Increased rest-phase overconsumption in wild-type mice fed on obesogenic diet.** All data (mean +/- SEM) from wild-type mice fed with normal chow (NC, white) and high-fat diet (HFD, grey) for 10 weeks. **(A)** Body weight development. **(B)** Comparative daily profiles of diet intake (n=12). **(C)** Overconsumption (n=6-12). Circa_single_mixed rhythmicity analysis in the case of repeated measurement analysis. R indicates that the dataset is rhythmic (p < 0.05). One-way RM ANOVA with Sidak's multiple comparation test. **p*<0.05, ***p*<0.01, ****p*<0.001, *****p*<0.0001.


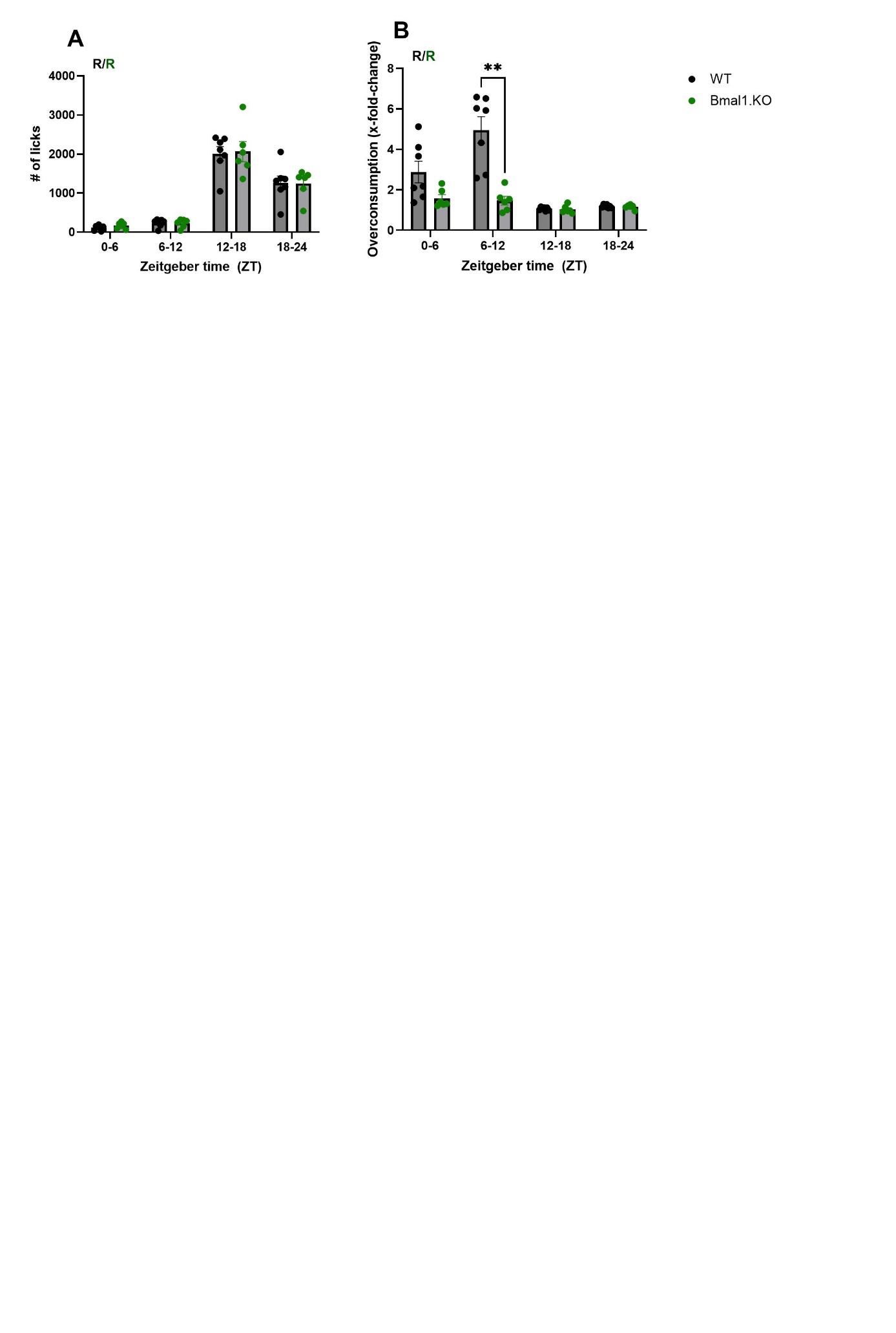


**Figure S2. Loss of clock function (in *Bmal1.KO* mice) alters leptin gating in the VTA and blunts hedonic intake rhythms.** All data (mean +/- SEM) from wild-type (WT, black) and *Bmal1.*KO mice (HFD, grey) for 10 weeks. **(A)** Comparative daily profiles of daily water intake (n=6-7). **(B)** Sucrose overconsumption (n=6-7). R and N indicate whether the dataset is rhythmic (p < 0.05) or non-rhythmic (p > 0.05). 2-way RM ANOVA with Sidak's multiple comparation test and Circa_single_mixed rhythmicity analysis in the case of repeated measurement analysis. **p*<0.05, ***p*<0.01, ****p*<0.001, *****p*<0.0001.


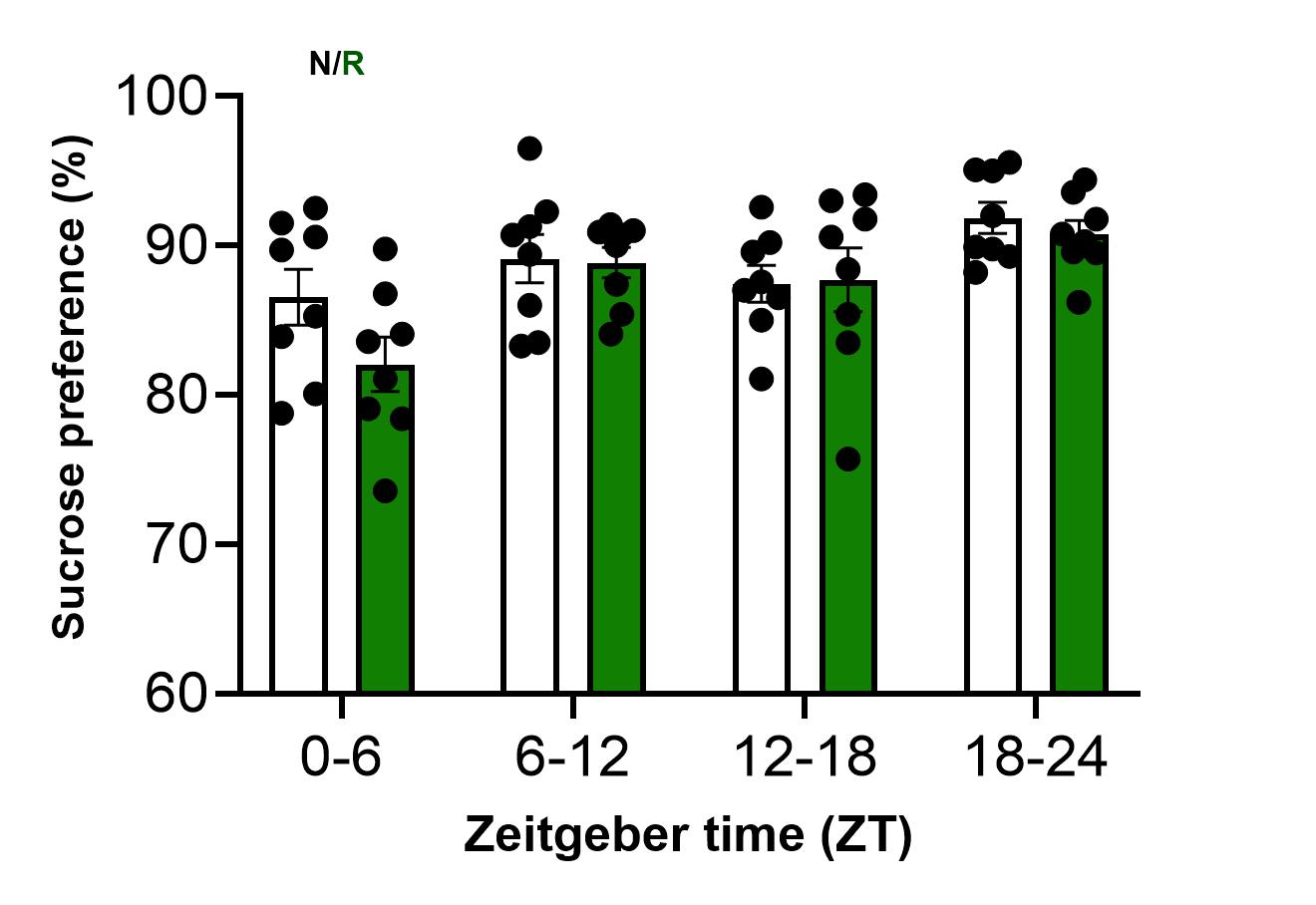


**Figure S3. Loss of clock function (in *Per1/2* mice) does not affect sucrose preference.** All data from *Per1/2* mice (green) and controls (wild-type, WT, white). Sucrose preference (n=8). N indicates that the dataset is non-rhythmic (p > 0.05). 2-way RM-ANOVA with Sidak's multiple comparation test and Circa_single_mixed rhythmicity analysis. *p< 0.05, ** p<0.01, ***p<0.001.


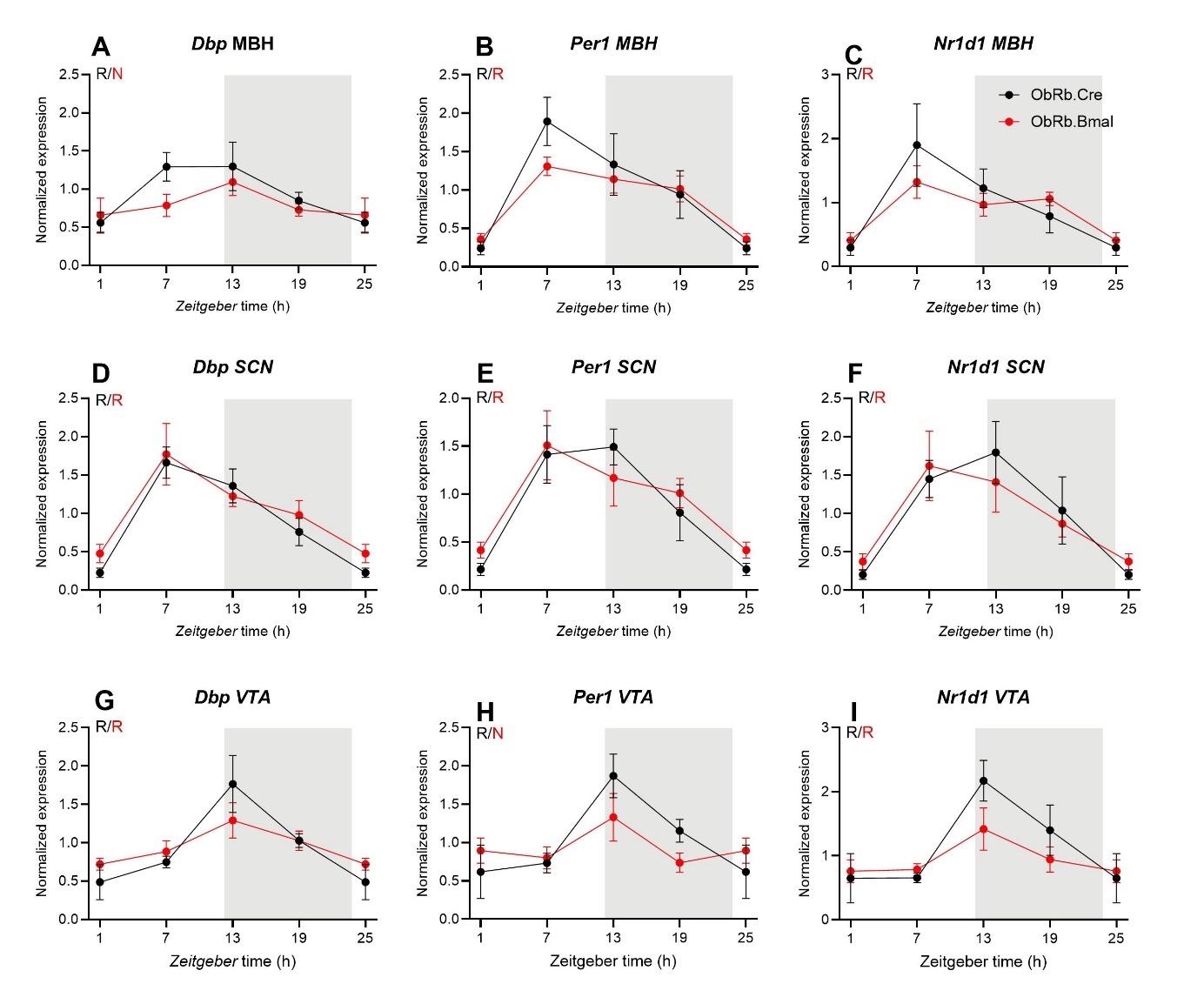


**Figure S4. *Dbp, Per1, Nr1d1* mRNA rhythms in *ObRb.Bmal1* mice** in mediobasal hypothalamus (MBH, n=4; **A-C**) suprachiasmatic nucleus (SCN, n=4; **D-F**) and ventral tegmental area (VTA, n=4; **G-I**). 2way-ANOVA and CircaCompare rhythmicity analysis. R and N indicate whether the dataset is rhythmic (p < 0.05) or non-rhythmic (p > 0.05). qPCR data analysis.


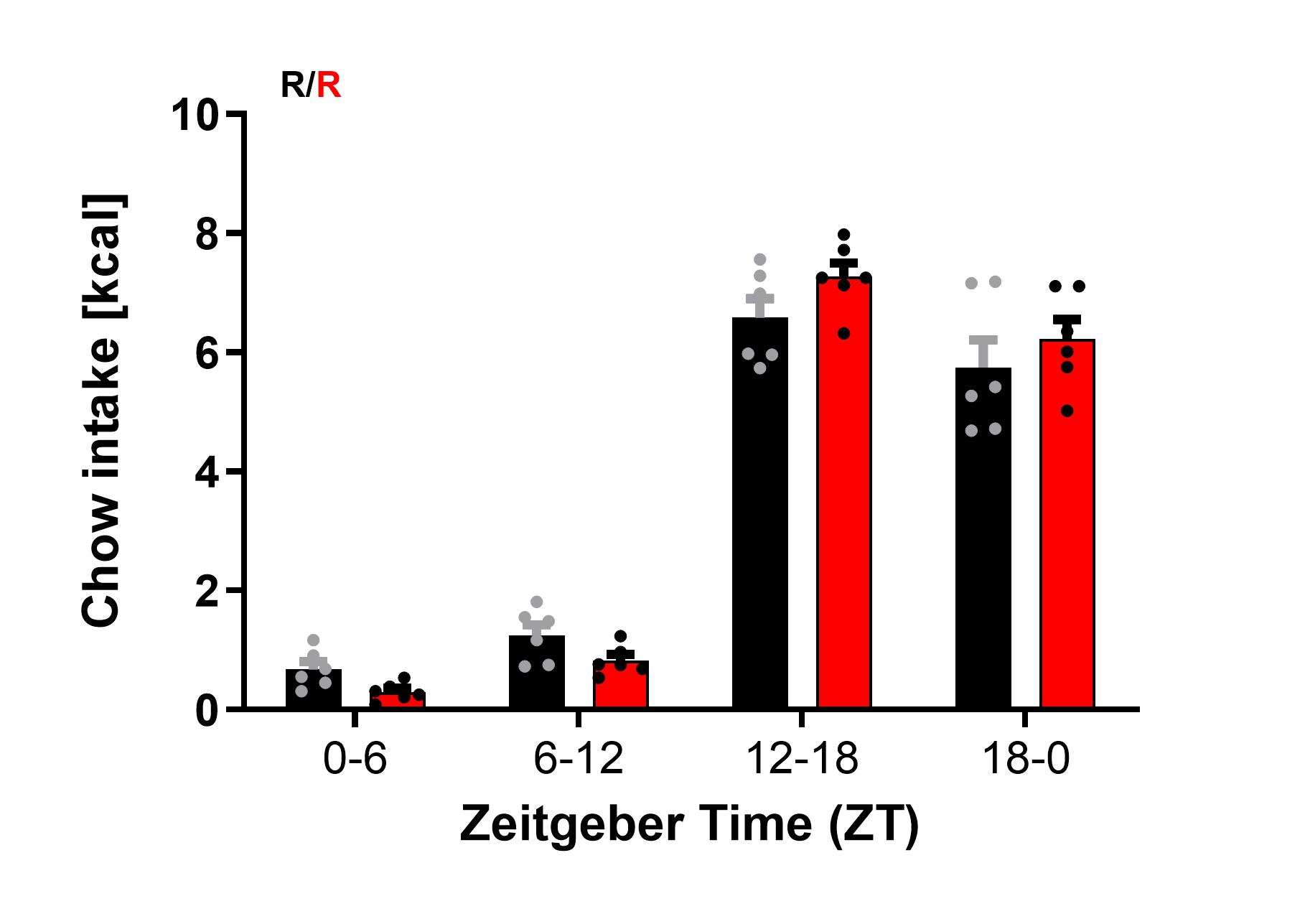


**Figure S5. Similar energy intake in obRb.Cre (black) and obRb.Bmal (red****).** Rhythmic chow intake over 24h in LD condition (n=6). R indicate that the dataset is rhythmic (p < 0.05). 2way RM-ANOVA with Sidak's multiple comparation test and Circa_single_mixed rhythmicity analysis. *p< 0.05, ** p<0.01, ***p<0.001.


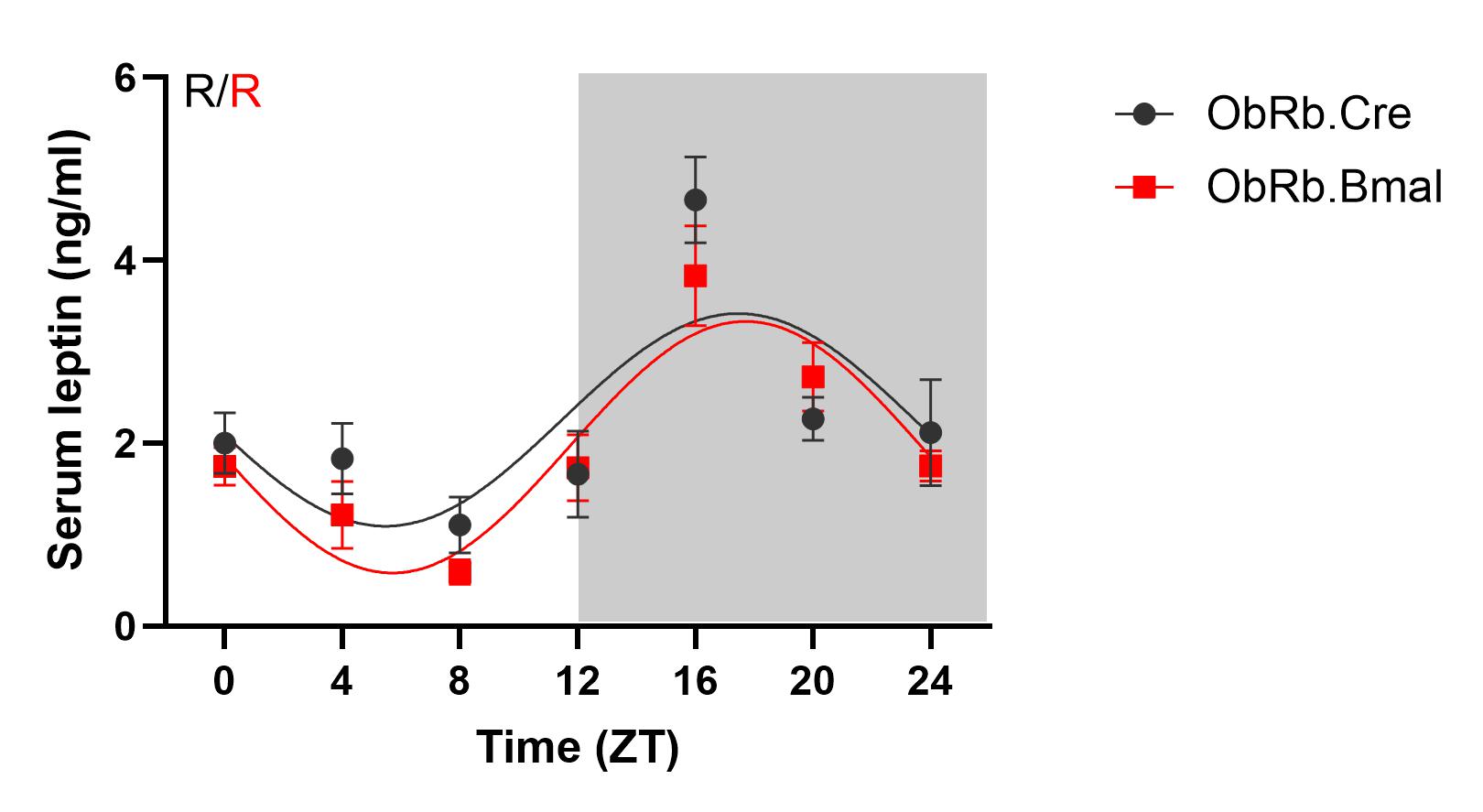


**Figure S6 Serum Leptin profile wild-type and ObRb.Bmal mice.** Rhythmic leptin secretion over 24h in LD condition (n=6). R indicate that the dataset is rhythmic (p < 0.05). 2way ANOVA with Sidak's multiple comparation test and CircaCompare rhythmicity analysis. R indicates that the dataset is rhythmic (p < 0.05)


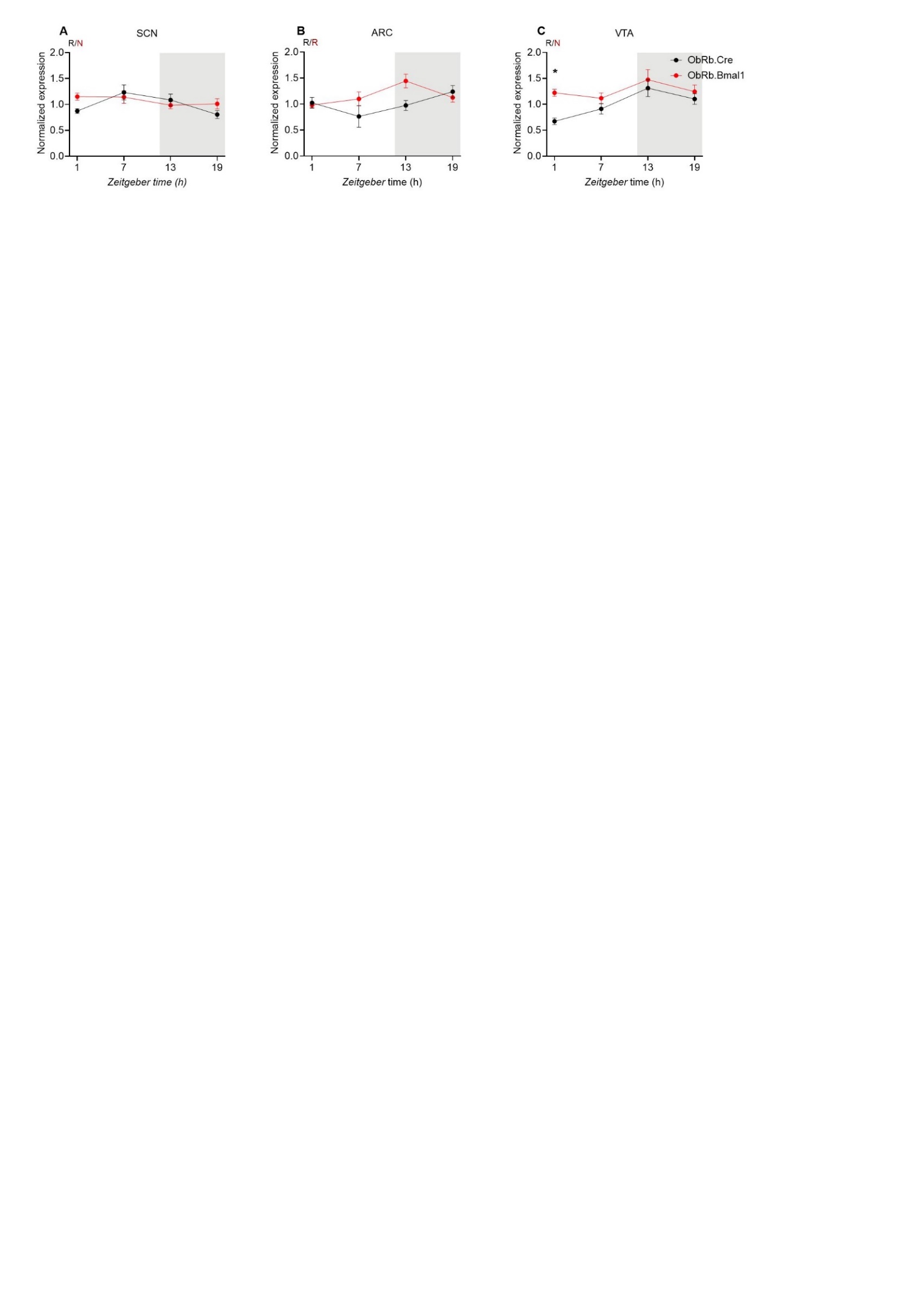


**Figure S7. Blunted leptin receptor gene expression rhythms in ObRb.Bmal1 KO mice** in SCN **(A)**, ARC **(B)** and VTA **(C)**. All qPCR assay data from ObRb.Bmal1 (red) and controls (ObRb.Cre, black). R and N indicate whether the dataset is rhythmic or non-rhythmic, respectively. R and N indicate whether the dataset is rhythmic (p < 0.05) or non-rhythmic (p > 0.05). 2-way ANOVA with Sidak's multiple comparation test and CircaCompare rhythmicity analysis. *p<0.05, **p<0.01, ***p<0.001, ****p<0.0001.


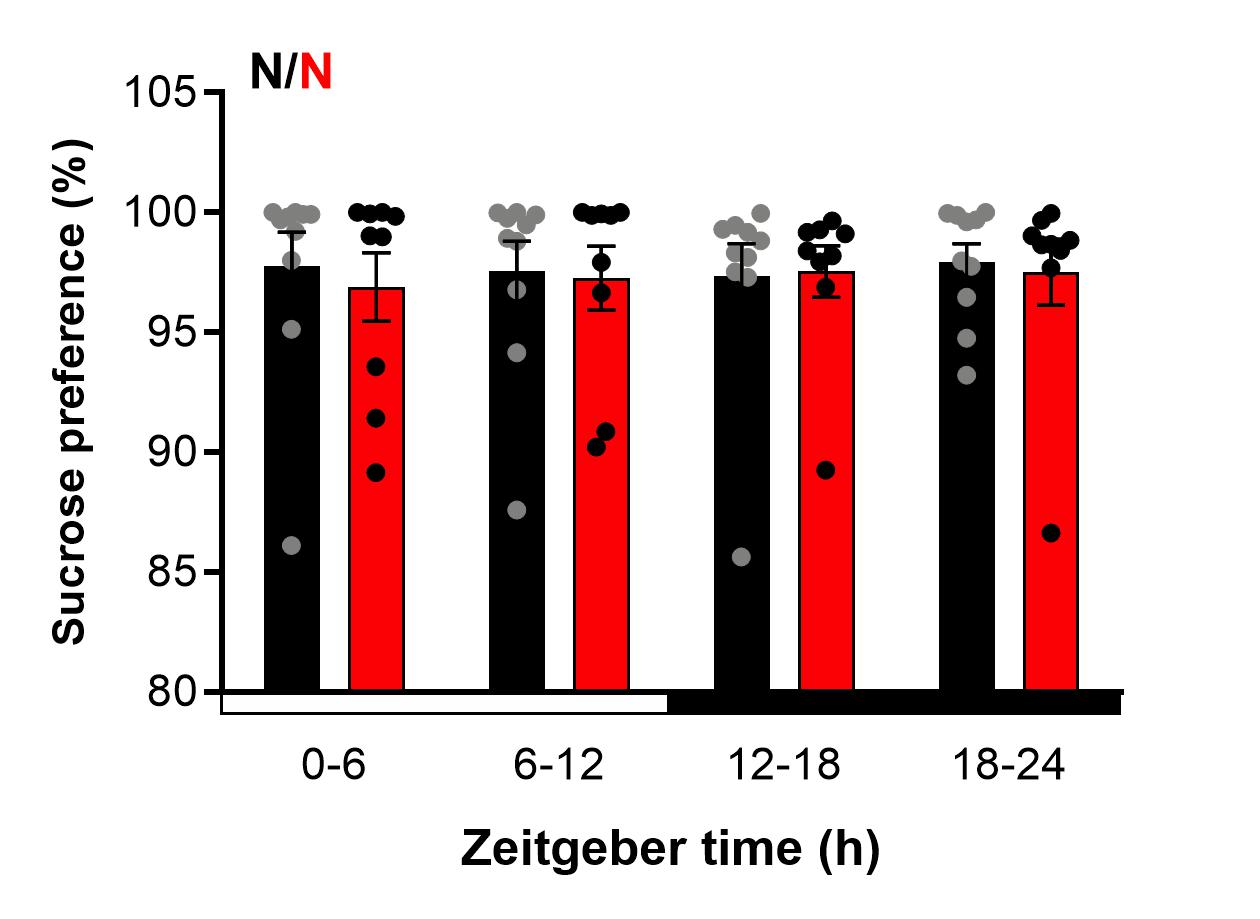


**Figure S8. Similar sucrose preference in** **ObRb.Bmal mice (red) and control** **ObRb.Cre (black).** Preference Sucrose (n=10). N indicate that dataset is non-rhythmic (p > 0.05). 2way RM-ANOVA with Sidak's multiple comparation test and Circa_single_mixed rhythmicity analysis . 2way RM-ANOVA. *p< 0.05, ** p<0.01, ***p<0.001.

**Table S1. Summary of the statistic analysis.**

| Figure | Test | Result |
| --- | --- | --- |
| Fig. 1A | Student's t-tests | *t*_10_=25.08, *p*<0.0001 |
| Fig. 1B | Student's t-tests | *t*_11_=5.94, *p*<0.0001 |
| Fig. 1C | Mixed effect analysis (repeated measurements) | **Interaction:** *F*_3,86_= 1.032, *p*=0.38; **time:** *F*_2.327,66.72_=11, *p*<0.0001;  **genotypes:** *F*_1,86_=51.99, *p*<0.0001 |
| Fig. 1C | Circa_single_mixed rhythmicity analysis | **Rhythmicity:** ***ob/+*** *p*<0.0001, ***ob/ob*** *p*<0.0001 |
| Fig. 1D | Student's t-tests | *t*_11_=2.51, *p*=0.02 |
| Fig. 1E | Student's t-tests | *t*_9_=1.23, *p*=0.24 |
| Fig. 1F | 2way RM-ANOVA | **Log ^10^ data, Interaction:** *F*_3,60_= 0.9216, *p*=0.43; **time:** *F*_1.559, 31.17_=76.18, *p*<0.0001; **genotypes:** *F*_1,20_=1.18, *p*=0.28 |
| Fig. 1F | Circa_single_mixed rhythmicity analysis | **Rhythmicity:** ***ob/ob*** *p*<0.0001, ***ob/+*** *p*<0.0001 |
| Fig. 1G | Student's t-tests | *t*_9_=0.05, *p*=0.95 |
| Fig. 1H | 2way RM-ANOVA | **Interaction** *F*_3,63_= 0.8402, *p*=0.47; **time:** *F*_3,63_=2.73, *p*=0.051;  **genotypes:** *F*_1,21_=0.28, *p*=0.60 |
| Fig. 1H | Circa_single_mixed rhythmicity analysis | **Rhythmicity:** ***ob/+*** *p*=0.22, ***ob/ob*** *p=*0.8 |
| Fig. 1I | Mixed effect analysis (repeated measurements) | **Log ^10^ data, Interaction:** *F*_3,58_= 3.01, *p*=0.03; **time:** *F*_3,58_=11.89, *p*<0.0001; **genotypes:** *F*_1,21_=19.35, *p=*0.0003 |
| Fig. 1I | Circa_single_mixed rhythmicity analysis | **Rhythmicity:** ***ob/+*** *p*<0.0001, ***ob/ob*** *p*<0.03 |
| Fig. 2A | 2way RM-ANOVA | **Interaction:** *F*_3,42_=0.36, *p*=0.77; **time:** *F*_1.621,22.70_=62.02, *p*<0.0001; **genotypes:** *F*_1,14_=1.38, *p*=0.25 |
| Fig. 2A | Circa_single_mixed rhythmicity analysis | **Rhythmicity:** **WT** *p*<0.0001, **Per1/2** *p*<0.0001 |
| Fig. 2B | 2way RM-ANOVA | **Interaction:** *F*_3,42_=3.14, *p*=0.03; **time:** *F*_3,42_=6.98, *p*=0.0006; **genotypes**: *F*_1,14_=12.12, *p*=0.003 |
| Fig. 2B | Circa_single_mixed rhythmicity analysis | **Rhythmicity:** **WT** *p=*0.0002, **Per1/2** *p*=0.60 |
| Fig. 2C | 2way-ANOVA | **Interaction:** *F*_1,16_=2.089, *p*=0.16; **treatment:** *F*_1,16_=38.99, *p*<0.0001; **genotype:** *F*_1,16_=0.24, *p*=0.66 |
| Fig. 2D | 2way-ANOVA | **Interaction:** *F*_1,16_=0.99, *p*=0.33; **treatment:** *F*_1,16_=61.34, *p*<0.0001; **genotype:** *F*_1,16_=4.57, *p*=0.04 |
| Fig. 2E | 2way-ANOVA | **Interaction:** *F*_1,16_=4.49, *p*=0.05; **treatment:** *F*_1,16_=19.83 *p*=0.0004; **genotype:** *F*_1,16_=0.18, *p*=0.67 |
| Fig. 2F | 2way-ANOVA | **Interaction:** *F*_1,16_=1.20, *p*=0.28; **treatment:** *F*_1,16_=4.74, *p*=0.04; **genotype:** *F*_1,16_=12.37, *p*=0.002 |
| Fig. 3G | 2way-ANOVA | **Interaction:** *F*_3,24_=9.61, *p*=0.0002; **time:** *F*_3,24_=18.64, *p*<0.0001; **genotypes:** *F*_1,24_=10.16, *p*=0.004 |
| Fig. 3G | CircaCompare rhythmicity analysis | **Rhythmicity:** **WT** *p*<0.0001, **Per1/2** *p*=0.08 |
| Fig. 3H | 2way-ANOVA | **Interaction:** *F*_3,24_=12.42, *p*<0.0001; **time:** *F*_3,24_=8.62, *p*=0.0005; **genotypes:** *F*_1,24_=4.32, *p*=0.048 |
| Fig. 3H | CircaCompare rhythmicity analysis | **Rhythmicity:** **WT** *p*<0.0001, **Per1/2** *p*=0.07 |
| Fig. 3I | 2way-ANOVA | **Interaction:** *F*_3,24_=12.72, *p*<0.0001; **time:** *F*_3,24_=19.27, *p*<0.0001; **genotypes:** *F*_1,24_=2.43, *p*=0.13 |
| Fig. 3I | CircaCompare rhythmicity analysis | **Rhythmicity:** **WT** *p*<0.0001, **Per1/2** *p*=0.1 |
| Fig. 4A | Student's t-tests | **ARC** *t*_10_=2.86, *p*=0.01, **VTA** *t*_10_=3.47, *p*=0.006, **SCN** *t*_10_=0.76, *p*=0.46 |
| Fig. 4B | Student's t-tests | *t*_2_=4,671, *p*=0.04 |
| Fig.4E | Student's t-tests | *t*_9_=2.30, *p*=0.04 |
| Fig. 4F | Student's t-tests | **DD, active phase** *t*_9_=2.17, **LL, inactive phase** *p*=0.05 *t*_9_=0.93, *p*=0.37 |
| Fig. 4G | 2way-ANOVA | **Interaction:** *F*_1,16_=3.405, *p*=0.08; **time:** *F*_1,16_=0.5941, *p*=0.45; **genotypes:** *F_1_*_,16_=15.95, *p*<0.001 |
| Fig. 4H | 2way-ANOVA | **Interaction:** *F*_1,16_=0.22, *p*=0.6432; **time:** *F*_1,16_=0.5393, *p*=0.4734; **genotypes:** *F_1_*_,16_=0.9248, *p*<0.35 |
| Fig. 5A | 2way RM-ANOVA | **Interaction:** *F*_11,368_=5.09, *p*<0.0001, **time:** *F*_1.588,53.14_=829, *p*<0.0001; **genotypes:** *F*_1,38_=2.21, *p*=0.14 |
| Fig. 5D | Student's t-tests | *t*_10_=0.97, *p*=0.35, *t*_10_=3.08, *p*=0.01 |
| Fig. 5E | Student's t-tests | *t*_10_=0.26, *p*=0.79 *t*_10_=0.07, *p*=0.94 |
| Fig. 5F | 2way RM-ANOVA | **Interaction:** *F*_3,12_=0.33, *p*=0.80; **time:** *F*_1.75,7.03_=3.12, *p*=0.11; **genotypes:** *F*_1,4_=1.76, *p*=0.25 |
| Fig. 5G | 2way RM-ANOVA | **Interaction:** *F*_23,138_=0.80, *p*=0.72; **time:** *F*_3.37,20.27_=18.24, *p*<0.0001; **genotypes:** *F*_1,6_=0.006, *p*=0.93 |
| Fig. 5H | Student's t-tests | **Lean mass** *t*_11_=2.08, *p*=0.06, **body fat** *t*_11_=1.97, **free fluid** *p*=0.07, *t*_11_=1.86, *p*=0.08 |
| Fig. 5I | 2way RM-ANOVA | **Interaction:** *F*_3,72_=0.22, *p*=0.88; **time:** *F*_3,72_=27.46, *p*<0.0001; **genotypes:** *F*_1,72_=1.26, *p*=0.26; |
| Fig. 5I | Circa_single_mixed rhythmicity analysis | **Rhythmicity:** ***ObRb.Cre*** *p*<0.0001, ***ObRb.Bmal1*** *p*<0.0001 |
| Fig. 5J | Mixed effect analysis (repeated measurements) | **interaction:** *F*_3,50_=3.08, *p*=0.03, **time:** *F*_1.032,17.20_=7.66, *p*=0.01; **genotypes:** *F*_1,18_=2.59, *p*=0.12; |
| Fig. 5J | Circa_single_mixed rhythmicity analysis | **Rhythmicity:** **ObRb.Cre** *p*=0.02, ***ObRb.Bmal1*** *p*=0.01 |
| Fig. 6C | Student's t-tests | *t*_20_=2.09, *p*=0.04 |
| Fig. 6D | Student's t-tests | *t*_12_=0.56, *p*=0.58 |
| Fig. 6E | Student's t-tests | **Chocolate:** *t*_20_=2.65, *p*=0.01 **breeding diet:** *t*_16_=1.08, *p*=0.29 |
| Fig. 6F | Student's t-tests | **chocolate:** *t*_12_=1.11, *p*=0.28, **breeding diet:** *t*_15_=1.73, *p*=0.10 |
| Fig. 6G | 2way-ANOVA | **interaction:** *F*_1,14_=6.63, *p*=0.02, **time:** *F*_1,14_=14.58, *p*=0.001; **genotypes:** *F*_1,14_=0.15, *p*=0.70 |
| Fig. 6H | 2way-ANOVA | **interaction:** *F*_1,10=_19.99, *p*=0.001, **time:** *F*_1,10_=5.08, *p*=0.04; **genotypes:** *F*_1,10_=1.17, *p*=0.30 |
| Fig. 7A | 2way RM-ANOVA | **interaction:** *F*_5,70=_27.58, *p*<0.0001, **time:** *F*_2.205,30.87_=225.9, *p*<0.0001; **genotypes:** *F*_1,14_=71.44, *p*<0.0001 |
| Fig. 7B | 2way RM-ANOVA | **interaction:** *F1*_,14=_27.20, *p*=0.0001, **time:** *F*_1,14_=30.86, *p*=0.0001; **genotypes:** *F*_1,14_=28.47, *p*=0.001 |
| Fig. 7C | 2way RM-ANOVA | **interaction:** *F*_5,70=_3.95, *p*=0.003, **time:** *F*_5,70_=494.4, *p*<0.001; **genotypes:** *F*_1,14_=6.87, *p*=0.02 |
| Fig. 7D | 2way RM-ANOVA | **interaction:** *F*_1,14=_5.87, *p*=0.03, **time:** *F*_1,14_=15.63, *p*<0.001; **genotypes:** *F*_1,14_=16.63, *p*=0.001 |
| Fig. 7E | 2way-ANOVA | **interaction:** *F*_1,28_=100.7, *p*=0.001, **diet:** *F*_1,28_=27.59, *p*<0.001; **genotypes:** *F*_1,28_=27.59, *p*=0.001 |
| Fig. S1A | Student's t-tests | *t_18_*=9.527, *p*<0.001 |
| Fig. S1B | One-way RM-ANOVA | *F*_2,349, 21,14_ = 43.55, P<0.0001 |
| Fig. S1B | Circa_single_mixed rhythmicity analysis | **Rhythmicity:** **HFD** *p*<0.0001 |
| Fig. S1C | One-way RM-ANOVA | *F* _2.022, 18.20_ = 31.16, P<0.0001 |
| Fig. S1C | Circa_single_mixed rhythmicity analysis | **Rhythmicity:** **HFD** *p*<0.0001 |
| Fig. S2A | 2way RM-ANOVA | **interaction:** *F*_3,33_=0.030, *p*=0.0.99; **time:** *F*_1.507,16.57_=69.17, *p*<0.0001; **genotypes:** *F*_1,11_=0.11, *p*=0.74 |
| Fig. S2A | Circa_single_mixed rhythmicity analysis | **Rhythmicity:** **WT** *p*<0.0001, ***Bmal1 KO*** *p*<0.0001 |
| Fig. S2B | 2way RM-ANOVA | **interaction:** *F*_3,33_=14.14, *p*=0.0001; **time:** *F*_1.765,19.41_=21.78, *p*=0.0001; **genotypes:** *F*_1,11_=16.44 *p*=0.001 |
| Fig. S2B | Circa_single_mixed rhythmicity analysis | **Rhythmicity:** **WT** *p*<0.0001, ***Bmal1 KO*** *p*=0.009 |
| Fig. S3 | 2way RM-ANOVA | **interaction:** *F*_3,14_=0.9806, *p*=0.41; **time:** *F*_2.172,30.41_=7.338, *p*=0.0002; **genotypes:** *F*_1,14_=1.74, *p*=0.20 |
| Fig. S3 | Circa_single_mixed rhythmicity analysis | **Rhythmicity:** **WT** *p=0.*21, ***Per1/2*** *p*=0.02 |
| Fig. S4A | 2way-ANOVA | **interaction:** *F*_4,30_=0.9153, *p*=0.46; **time:** *F*_4,30_=3.969, *p*=0.01; **genotypes:** *F*_1,30_=1.156, *p*=0.29 |
| Fig. S4A | CircaCompare rhythmicity analysis | **Rhythmicity:** ***ObRb.Cre*** *p=*0.001, ***ObRb.Bmal*** *p*>0.05 |
| Fig. S4B | 2way-ANOVA | **interaction:** *F*_4,30_=0.9998, *p*=0.42; **time:** *F*_4,30_=14.46, *p*=0.0001; **genotypes:** *F*_1,30_=0.4745, *p*=0.49 |
| Fig. S4B | CircaCompare rhythmicity analysis | **Rhythmicity:** ***ObRb.Cre* =**0.0007, ***ObRb.Bmal*** *p*=0.0001  **Amplitude estimate:** ***ObRb.Cre*** *p=*0.79, ***ObRb.Bmal*** *p*=0.47 |
| Fig. S4C | 2way-ANOVA | **interaction:** *F*_4,30_=0.7952, *p*=0.53; **time:** *F*_4,30_=7.69, *p*=0.0002; **genotypes:** *F*_1,30_=0.1607, *p*=0.69 |
| Fig. S4C | CircaCompare rhythmicity analysis | **Rhythmicity:** ***ObRb.Cre*** *p=*0.004, ***ObRb.Bmal*** *p*=0.007  **Amplitude estimate:** ***ObRb.Cre*** *p=*0.78, ***ObRb.Bmal*** *p=*0.37 |
| Fig. S4D | 2way-ANOVA | **interaction:** *F*_4,30_=0.3641, *p*=0.83; **time:** *F*_4,30_=19.04, *p*=0.0001; **genotypes:** *F*_1,30_=1.306, *p*=0.26 |
| Fig. S4D | CircaCompare rhythmicity analysis | **Rhythmicity:** ***ObRb.Cre*** *p*<0.0001, ***ObRb.Bmal*** *p*=0.001  **Amplitude estimate:** ***ObRb.Cre*** *p=*0.77, ***ObRb.Bmal*** *p=*0.59 |
| Fig. S4E | 2way-ANOVA | **interaction:** *F*_4,30_=0.5439, *p*=0.70; **time:** *F*_4,30_=12.43, *p*=0.0001; **genotypes:** *F*_1,30_=0.3011, *p*=0.58 |
| Fig. S4E | CircaCompare rhythmicity analysis | **Rhythmicity:** ***ObRb.Cre*** *p*<0.0001, ***ObRb.Bmal*** *p*=0.003  **Amplitude estimate:** ***ObRb.Cre*** *p=*0.73, ***ObRb.Bmal*** *p=*0.50 |
| Fig. S4F | 2way-ANOVA | **interaction:** *F*_4,30_=0.4016, *p*=0.80; **time:** *F*_4,30_=9.857, *p*<0.0001; **genotypes:** *F*_1,30_=0.0023, *p*=0.96 |
| Fig. S4F | CircaCompare rhythmicity analysis | **Rhythmicity:** ***ObRb.Cre*** *p*<0.0001, ***ObRb.Bmal*** *p*=0.001  **Amplitude estimate:** ***ObRb.Cre*** *p=*0.85, ***ObRb.Bmal*** *p*=0.68 |
| Fig. S4G | 2way-ANOVA | **interaction:** *F*_4,30_=0.1.221, *p*=0.32; **time:** *F*_4,30_=8.306, *p*=0.0001; **genotypes:** *F*_1,30_=0.0422, *p*=0.83 |
| Fig. S4G | CircaCompare rhythmicity analysis | **Rhythmicity:** ***ObRb.Cre*** *p=*0.0002, ***ObRb.Bmal*** *p*=0.002  **Amplitude estimate:** ***ObRb.Cre*** *p=*0.62, ***ObRb.Bmal*** *p*=0.28 |
| Fig. S4H | 2way-ANOVA | **interaction:** *F*_4,30_=1.359, *p*=0.27; **time:** *F*_4,30_=4.815, *p=*0.004; **genotypes:** *F*_1,30_=0.2036, *p*=0.65 |
| Fig. S4H | CircaCompare rhythmicity analysis | **Rhythmicity:** ***ObRb.Cre*** *p=*0.001, ***ObRb.Bmal*** *p*>0.05 |
| Fig. S4I | 2way-ANOVA | **interaction:** *F*_4,30_=1.083, *p*=0.38; **time:** *F*_4,30_=5.851, *p=*0.001; **genotypes:** *F*_1,30_=0.9532, *p*=0.33 |
| Fig. S4I | CircaCompare rhythmicity analysis | **Rhythmicity:** ***ObRb.Cre*** *p=*0.0001, ***ObRb.Bmal*** *p*=0.02  **Amplitude estimate:** ***ObRb.Cre*** *p=*0.79, ***ObRb.Bmal*** *p*=0.30 |
| Fig. S5 | 2way RM-ANOVA | **interaction:** *F*_3,30_=2.45, *p*=0.08; **time:** *F*_1.953,19.53_=323.8, *p*=0.001; **genotypes:** *F*_1,10_=0.23, *p*=0.63 |
| Fig. S5 | Circa_single_mixed rhythmicity analysis | **Rhythmicity:** ***ObRb.Cre*** *p*<0.0001, ***ObRb.Bmal*** *p*<0.0001 |
| Fig. S6 | 2way ANOVA | **interaction:** *F*_6,71_=0.67, *p*=0.66; **time:** *F*_6, 71_=16.22, *p<*0.0001; **genotypes:** *F*_1,71_=2.213, *p*=0.14 |
| Fig. S6 | CircaCompare rhythmicity analysis | **Rhythmicity:** ***ObRb.Cre*** *p>0.*0001, ***ObRb.Bmal*** *p*>0.0001 |
| Fig. S7A | 2way-ANOVA | **interaction:** *F*_3,24_=2.051, *p*=0.13; **time:** *F_3.24_*=2.806, *p*=0.06; **genotypes:** *F*_1,24_=1.13, *p*=0.29 |
| Fig. S7A | CircaCompare rhythmicity analysis | ***ObRb.Cre*** *p=*0.007, ***ObRb.Bmal*** *p=*0.17 |
| Fig. S7B | 2way-ANOVA | **interaction:** *F*_3,24_=2.611, *p*=0.07; **time:** *F_3.24_*=2.381, *p*=0.09; **genotypes:** *F*_1,24_=3.42, *p*=0.07 |
| Fig. S7B | CircaCompare rhythmicity analysis | ***obRb.Cre*** *p=0.*02, ***obRb.Bmal*** *p=0.*01 |
| Fig. S7C | 2way-ANOVA | **interaction:** *F*_3,24_=1.21, *p*=0.32; **time:** *F_3.24_*=5.14, *p*=0.006; **genotypes:** *F*_1,24_=9.16, *p*=0.005 |
| Fig. S7C | CircaCompare rhythmicity analysis | ***ObRb.Cre*** *p=*0.008, ***ObRb.Bmal*** *p=*0.14 |
| Fig. S8 | 2way ANOVA | **interaction:** *F*_3,51_=0.09, *p*=0.96; **time:** *F*_1.811,30.78_=0.05, *p*=0.93; **genotypes:** *F*_1,17_=0.08, *p*=0.78 |
| Fig. S8 | Circa_single_mixed rhythmicity analysis | **Rhythmicity:** ***ObRb.Cre*** *p>0.*05, ***ObRb.Bmal*** *p*>0.05 |
